# Advanced Constitutive Modeling of the Thixotropic Elasto-Visco-Plastic Behavior of Blood: Description of the Model and Rheological Predictions

**DOI:** 10.3390/ma13184184

**Published:** 2020-09-20

**Authors:** Konstantinos Giannokostas, Pantelis Moschopoulos, Stylianos Varchanis, Yannis Dimakopoulos, John Tsamopoulos

**Affiliations:** Laboratory of Fluid Mechanics and Rheology, Department of Chemical Engineering, University of Patras, 26504 Patras, Greece; giannoko@upatras.gr (K.G.); pmoschopoulos@chemeng.upatras.gr (P.M.); varchanis@chemeng.upatras.gr (S.V.); tsamo@chemeng.upatras.gr (J.T.)

**Keywords:** TEVP, constitutive modeling, blood, hemorheology, thixotropy, elastoviscoplastic, blood viscoelasticity, shear rheometry, LAOS, extensional rheometry, thixotropic index, blood relaxation time, personalize parametrization

## Abstract

This work focuses on the advanced modeling of the thixotropic nature of blood, coupled with an elasto-visco-plastic formulation by invoking a consistent and validated model for TEVP materials. The proposed model has been verified for the adequate description of the rheological behavior of suspensions, introducing a scalar variable that describes dynamically the level of internal microstructure of rouleaux at any instance, capturing accurately the aggregation and disaggregation mechanisms of the RBCs. Also, a non-linear fitting is adopted for the definition of the model’s parameters on limited available experimental data of steady and transient rheometric flows of blood samples. We present the predictability of the new model in various steady and transient rheometric flows, including startup shear, rectangular shear steps, shear cessation, triangular shear steps and LAOS tests. Our model provides predictions for the elasto-thixotropic mechanism in startup shear flows, demonstrating a non-monotonic relationship of the thixotropic index on the shear-rate. The intermittent shear step test reveals the dynamics of the structural reconstruction, which in turn is associated with the aggregation process. Moreover, our model offers robust predictions for less examined tests such as uniaxial elongation, in which normal stress was found to have considerable contribution. Apart from the integrated modeling of blood rheological complexity, our implementation is adequate for multi-dimensional simulations due to its tensorial formalism accomplished with a single time scale for the thixotropic effects, resulting in a low computational cost compared to other TEVP models.

## 1. Introduction

Blood is a complex suspension of red blood cells (RBCs), white blood cells (WBCs) and platelets in an aqueous solution, the so-called plasma, containing dissolved proteins [1]. Although many works consider blood as a Newtonian fluid [2,3], in fact, it has a pronounced non-Newtonian character, mostly explained in terms of the ability of RBCs to aggregate/disaggregate, deform and align to flow [4,5,6,7]. At low shear rates, blood proteins enhance the formation of a complicated network made up of column-like red cell aggregates known as rouleaux, while at almost stasis, the rouleaux forms three-dimensional networks [8]. At higher shear rates, these structures tend to disintegrate, leading to a state where the red blood cells flow separately. The physical mechanism that drives the creation of rouleaux is not settled yet but two theories emerge as potential candidates, namely the bridging of fibrinogen [9,10] and the depletion theory [11]. This aggregation is a dynamic property of blood [12] promoting its non-Newtonian nature. The aforementioned behavior necessitates the use of sophisticated rheological models to capture adequately the rheological response of blood. 

In the last decades, investigation of hemorheology has stimulated a lot of attention to the complex nature of blood mostly due to the direct relevance of blood rheology to disease detection, treatment, and prevention. A number of diseases exhibit discernable hemorheological changes and vice versa, including sickle cell anemia, which is the most frequent genetic disease in the world, diabetes, hypertension, or heart disease [13,14,15] or connective tissue disease, blood hypercoagulability due to COVID-19 [16,17], arteriosclerosis, or diabetic blood [18,19,20]. An indicative example is the coagulation process, which initiates the formation of a fibrin network along with the clumping of red blood cells giving rise to enhanced elastic aspects [21]. The latter process becomes of significant importance when clotting of blood occurs [22,23], and the influence upon tissue is much more severe than when only the blood viscosity, which is another significant hemorheological parameter, is increased. It should be mentioned that during the clotting process, the elastic modulus varies with time by many orders of magnitude especially for a diabetic blood [24]. As a result, hemorheological measurements can potentially offer an individualized and straightforward method for detecting the presence of such diseases. However, using hemorheology as a diagnostic tool requires both data and accurate constitutive models for healthy and disease states. Of equal importance for advancing the engineering design of medical devices is the ability to simulate in silico the in vivo flow of blood, which requires accurate knowledge of the transient material properties of blood. 

Based on our previous discussion, it is perceivable that the rheological characterization of blood can be divided in two categories: steady state analysis and transient analysis. Initially, a significant effort focused on the steady-state shear rheometry, excluding phenomena such as the formation and dissolution of the rouleaux structures, which are placed at differing time scales, and are both flow-dependent. However, steady-state investigations should not be underestimated as they have given an insight into non-Newtonian characteristics and their association with blood flow. Blood possesses a distinct property according to which it flows as fluid if subjected to large enough stresses but behaves as a soft matter if the applied stress is below a critical value, giving rise to the experimentally measured blood yield stress [25,26]. The latter is an essential component of its non-Newtonian nature, stemming explicitly from the creation of rouleaux. Its role is most clearly evaluated under steady-state shear flow conditions. Experimental evidence of the exhibition of the yield stress has been provided by many investigations [6,25,27,28] leading to viscoplastic modeling of blood (VP) [29]. Additionally, the well-known shear thinning behavior of blood has been described by a plethora of investigations [30,31] in various hemodynamical conditions as well as the existence of a critical hematocrit associated with blood yield stress and the transition from non-Newtonian to Newtonian flow in high shear rates [32].

However, blood possesses also a non-negligible elastic nature, as shown experimentally [33,34]. Apparently, this is an inherent property of blood, [35,36] and it can be associated with the lipidic bilayer membrane of RBCs and hemorheological variables such as hematocrit and fibrinogen concentration [37,38]. Recently, Varchanis et al. [39] showed that the elastic nature of human blood plasma should not be neglected in regimes of intense shear. Particularly, important contributions to the experimental investigation of the viscoelasticity have been provided by Thurston [34], who performed oscillatory flow experiments in cylindrical tubes. Blood is also classified as a non-ideal yield-stress material due to its non-negligible elastic effect. Thus, the need to introduce the concept of elasto-visco-plastic (EVP) fluids is clear to describe materials that have elastic and plastic characteristics simultaneously. 

Although the findings of the aforementioned investigations are important, the inherent properties of blood are strongly time-dependent as well, and thus, blood is categorized as a thixotropic material. From a rheological perspective, blood is one of the most characteristic examples of a thixotropic fluid and allows for the evaluation of generalized thixotropy models for a unique and biologically relevant case. A thixotropic fluid is a fluid whose material properties (e.g., viscosity) is a function not only of the applied stress but also of the previous history of motion within the fluid [5,21,24,40,41]. The thixotropic nature of blood stems from the aggregation/disaggregation of rouleaux which is governed by its own time scales affected by the concentration of plasma proteins [42] and hematocrit [9]. In particular, the yield stress in dense, soft colloidal suspensions such as blood is typically attributed to an internal structure that develops, deforms, and decays in a way that depends critically not only on the current flow kinematics but also on its deformation history, thus giving rise to thixotropy [9,21,43,44]. Some of the first reports were those of Lacombe and Quemada [21,45], who focused on the description of these transient blood flow phenomena. One of the prominent experimental investigations is that of Bureau et al. [46] who systematically obtained hysteresis and step-up curves of pathological and physiological human blood. Here, we invoke the experimental work of McMillan et al. [47], who performed steady-state shear and rectangular shear-step tests which are capable to probe the elasto-thixotropic response of blood making our model robust and reliable. More recently, Armstrong et al. [48] and Horner et al. [49] conducted experiments in steady and transient rheometric flows. Except for the triangular step (hysteresis) experiments, the application of the oscillatory flow is commonly employed to probe the complex properties of blood. 

The rheological modeling of blood should consider all the above phenomena to predict a realistic behavior. Therefore, the focus has been shifting towards constitutive equations that incorporate plasticity, elasticity and thixotropy. The model of Owens and coworkers [50], which has been recently revised [51,52,53], was derived using ideas drawn from polymer network theory accounting for the agglomeration and deagglomeration of erythrocytes in healthy human blood at different shear rates. Although this model was subsequently applied to simple shear flows as well as to steady, oscillatory, and pulsatile flow in rigid vessels [54], it lacks explicit accounting for yield stress, the most important manifestation of the viscoplastic nature of blood. Additionally, Anand and Rajagopal [55] used a generalized Oldroyd-B model, which was developed in the context of the general thermodynamic framework of Rajagopal and Srinivasa [56]. They made use of a tensorial viscoelastic model and therefore do not explicitly take into account the viscoplastic nature of blood, although their work was found to agree with steady-state and transient experiments. Early efforts to investigate the thixotropy of blood were made by Apostolidis et al. [57], who based on a phenomenological model to incorporate thixotropy and viscoplasticity of blood, employed the Casson model, which is suitable for steady-state flows. They offered a systematic study of the rheology of blood in transient shear flows, based on a single scalar internal structural thixotropic parameter. We note that these models have primarily originated from the need for macroscopic modelling of materials composed by particles where phenomena such as the formation of agglomerates are pronounced [58,59]. Later it was suitably modified to exploit recent advances based on the kinematic hardening model of the elastic strain in plasticity theory of Dimitriou et al. [60]. Although this model was evaluated in transient and steady experiments, it lacks a tensorial form preventing its use in simulating multi-dimensional flows. Another remarkable theoretical investigation was made by Kaliviotis & Yianneskis [61] who presented a theoretical examination of the hypothesis that the network characteristics of red blood cell influence the mechanical properties of the blood. They developed an energy-rate based model, which incorporated network dynamics used to predict the transient behavior of blood suggesting that network characteristics play a significant role in the configuration of the blood viscosity. 

The coupled appearance of several elastic, plastic and thixotropic phenomena and the underlying mechanisms and internal variables in thixotropic elasto-visco-plastic (TEVP) materials necessitates the introduction of a large number of adjustable parameters to describe their rheology. The model proposed by Varchanis et al. [62] was suitable for highly concentrated suspensions, which exhibit shear thinning, thixotropy, plasticity and viscoelasticity. It is adequately modified in order to have a stress-controlled dynamic configuration of the structure parameter, accompanied by a reduction in fitting parameters. Our model consists of a set of eleven adjustable parameters, contrary to the original model, which in addition incorporates the kinematic hardening mechanism and consequently has extra parameters. We offer a consistent TEVP constitutive model that encompasses the most crucial characteristics of the aforementioned blood properties in order to validate experimental results satisfactorily and provide accurate predictions for steady-state flow regimes in microchannels. Our model is shown to produce results that agree well both with steady state and transient blood flow data [47]. Apart from the integrated modeling of blood rheological complexity, our implementation is adequate for multi-dimensional simulations due to its tensorial formalism, contrary to recent investigations of blood flow [57], which do not offer a tensorial form of their model. Another superior advantage is the single mode approach, meaning that the thixotropic effects follow a single time scale. A multimode thixotropic model is directly related to additional computational cost, which in the case of -two or -three-dimensional flows would be prohibitive. The single thixotropic time scale used in the present study, can yield satisfactory results and capture the behavior of blood, given that a proper rheological characterization has been performed. Our model provides an extensive predictive capability in steady and transient testing flow regimes and demonstrates rheological behavior, which is consistent with limited existing experimental data.

## 2. Constitutive Modelling

In this section, we outline the components of the constitutive model by invoking the work of Varchanis et al. [62], which has been based on Saramito’s model (SRM) [63] for EVP materials. Generally speaking, the starting point is the definition of the extra stress tensor τ which is composed of a purely Newtonian contribution, $\tau_{n}$, and a viscoelastic one, $\tau_{ve}$ as:

(1) $$\tau =\tau_{n}+\;\tau_{ve}$$

Our model accounts not only for the viscoelastic contribution of the RBCs but also for the viscoelasticity of blood plasma since it has been proved to present a significant non-Newtonian behavior because of the presence of plasma proteins [39,64]. Thus, there is no explicit Newtonian contribution $\left(\tau_{n}=0\right)$ as long as the solvent (plasma) is included in the viscoelastic term ($\tau_{ve}$).

Following the studies of Dimitriou and McKinley [60], Fraggedakis et al. [65], Stickel et al. [66], Dimitriou et al. [67], Clarion et al. [68] and Horner et al. [49], we assume that the total rate of deformation tensor:

(2) $$D=\frac{1}{2}\left[\left(\nabla u\right)+{\left(\nabla u\right)}^{T}\right]$$

is composed of an elastic contribution $D_{e}$ and a viscoplastic one denoted by $D_{vp}$ so that:

(3) $$D=\;D_{e}+\;D_{vp}$$

The elastic term of the total deformation rate tensor D which can be written as:

(4) $$D_{e}=\;\;\frac{1}{2\;G}\overset{\nabla }{\tau }$$

Considering the memory effects by introducing the upper-convected time derivative as in the majority of VE models, while G denotes the elastic modulus of the blood. For relatively low shear rates, the elastic aspects are pronounced, and the suspension is acting like a neo-Hookean solid. This can be justified by the appearance of the upper convected derivative into the elastic term. At the same time, for a Hookean solid, the linear relationship between strain and stress would be denoted by the introduction of the material derivative. For any tensor z, the upper convective time derivative is defined as:

(5) $$\overset{\nabla }{z}=\frac{\partial z}{\partial t}+u\;\cdot \nabla z-\;{\left(\nabla u\right)}^{T}\cdot z-z\;\cdot \left(\nabla u\right)$$

The effect of plasticity is introduced via multiplication of distinct functions and contributions given by the expression:

(6) $$D_{vp}=\;\;f\left[tr\left(\tau_{eff}\right)\right]\max\left(0,\frac{\sigma_{eff}-\;\tau_{y}}{2\;\eta_{t}\;\sigma_{eff}}\right)\tau_{eff}$$

Following the original TEVP model proposed by Varchanis et al. [62], $\tau_{eff}$ incorporates the back stresses which arise due to kinematic hardening (KH). The latter along with isotropic hardening (IH) are mechanisms related to variations in the material structure (e.g., dynamics of microparticles of a suspension, RBC aggregates). Also, they are very well known in the plasticity literature of solid mechanics. Primarily, the KH function is highlighted by flow reversal experiments, and it has been observed to be an inherent property of solids. Consequently, although KH could be a crucial component for a VP constitutive modelling, here we assume that it has negligible contribution, in the absence of flow reversal experiments and due to the belief that blood does not exhibit intense kinematic hardening, so as $\tau_{eff}=\;\tau_{ve}$. 

The von Mises criterion is essential for controlling the transition between the structured (e.g., rouleaux) and the destructured (e.g., individual RBCs) states which is introduced via a continuous max() function for any value of the magnitude of the effective stress $\sigma_{eff}$. This criterion is an ellipsoidal representation in stress space when the equal sign holds in the following, $\sigma_{eff}\;\geq \;k_{t}$. The effective stress is defined through the deviatoric part of the stress tensor as $\sigma_{eff}=\left|\tau_{eff}\right|=\;\sqrt{\left(\tau_{ve}{}^{D}:\tau_{ve}{}^{D}\right)/2}$, where the deviatoric part of any tensor z is defined as $z^{D}=z-\left(\frac{tr\left(z\right)}{3}\right)I$. The plasticity term is defined in accordance with the SRM model [63] multiplied with a nonlinear term which is a function of the trace of the viscoelastic stress tensor $\tau_{ve}$, as imposed by the linear Phan-Thien Tanner (PTT) function $f\left[tr\left(\tau_{ve}\right)\right]$. In contrast to the original model, here the PTT model bounds the extensional viscosity by an extra fitting parameter at high strain rates. Using the linear form of PTT function, which has been found to be the most suitable for this VE modeling, we exclude the contribution of shear thinning because this is suitable for exclusively VE consideration as argued by Varchanis et al. [69]. However, in our model shear thinning is mainly introduced via thixotropic viscosity $\eta_{t}$. Accordingly, the stress PTT function is defined as:

(7) $$f\left[tr\left(\tau_{ve}\right)\right]=\;1\;+\;\varepsilon_{PTT}\;\frac{tr\left(\tau_{ve}\right)}{G}$$

where $\varepsilon_{PTT}$ is a fitted parameter. For closure reasons of the model, we need to define equations that govern the evolution of the internal variables. Most of the investigations on thixotropic modelling incorporate a component of the structural statement, which is represented by a non-dimensional structure variable, *λ*. This parameter, in our case, reflects the current level of rouleaux formation within the blood sample, which changes over time. For simplicity, it varies in a predetermined range with limits corresponding to the two extreme cases regarding the level of the material structure. More specifically, when λ = 1, the blood is assumed to be in a fully structured state (jammed rouleaux), while when λ = 0, the blood is supposed to be completely unstructured (individual RBCs deform and flow with blood) and rheologically softer. Firstly, we present the equation governing the thixotropic structural changes within the blood. The time depended evolution of the structure (or rouleaux) parameter is then given by Wei et al. [59,70]:

(8) $$\frac{d\lambda }{dt}\;=k_{r}\;-\;k_{b}\;$$

The term $k_{r}$ in Equation (8) represents the rebuilding of the structure given by:

(9) $$k_{r}\;=\left(k_{1}+k_{2}\varphi^{n_{1}}\right)\left(1-\lambda \right)$$

which is caused either by Brownian collisions of individual RBCs, scaled with $k_{1}$, or by deformation scaled with $k_{2}$, while both contributions are proportional to the fraction of unstructured blood (e.g., individual RBCs) (1 – λ). On the contrary, $k_{b}$ refers to the breakdown which is assumed to grow proportionally to the shearing and the fraction of structured blood, *λ* as:

(10) $$k_{b}\;=k_{3}\varphi^{n_{2}}\lambda^{n_{3}}$$

where the $k_{3}$ parameter scales the impact of the viscoplastic rate of deformation on the breaking of rouleaux. Several different representative kinetic equations can be found in the literature and are summarized in the review of [70], while the «flow parameter» φ can be either deformation rate or stress controlled, or a combination of both. Contrary to Varchanis et al. [62], we have chosen to base the dependency of the level of structure explicitly on the stresses as also adopted by Wei et al. [59]. Consequently, our model incorporates a stress-controlled form given by:

(11) $$\varphi =\;\max\left(0,\sigma_{eff}-\;\tau_{y}\right)$$

Note that the rate-controlled (RC) and stress-controlled (SC) forms correspond to two similar, but different models. They make different constitutive assumptions about how flow conditions influence the thixotropic kinetics. 

In the absence of kinematic hardening (KH), the material possesses a yield stress that is denoted by $\tau_{y}$. Although in the original TEVP model [62], the last parameter depends on the magnitude of the deformation rate through λ, here for simplicity, we argue that the blood yield stress is not related to history effects, and thus, the impact of isotropic hardening (IH) on the yield stress is not taken into consideration. This contribution to the yield stress is most easily appreciated, representing the strength of the structural network due to bonds between its components. On the contrary, the plastic viscosity of the material $\eta_{t}$ is a thixotropy-dependent variable. Because of its dependence on λ, the current model is classified as nonideal thixotropic material. Finally, the variation of the plastic viscosity $\eta_{t}\left(\lambda \right)$ must be specified. Contrary to the simplest linear relationship proposed by Dimitriou et al. [60], we generalize the dependence of the rheological parameters on the structure variable and propose a simple relationship to include a nonlinearity, as suggested by Geri et al. [71] for these materials. From a mathematical perspective, we propose an expression able to control this nonlinearity based on the response of the TEVP material to flow. Thus, the dependence of the viscosity on λ is defined as:

(12) $$\eta_{t}\left(\lambda \right)=\eta_{0}\lambda^{m_{1}}$$

where $\eta_{0}$, $m_{1}$ are fitting parameters. Because of the bounded value of the structure parameter in the range of 0–1, plastic viscosity will attain values in a predetermined range ($\eta_{n}$, $\eta_{t,1}$] depending on the value of λ. The limit $\eta_{t,1}$ denotes the value of the parameter $\eta_{t}$ when the blood is in a fully structured state (λ = 1), while in almost fully unstructured case (λ~0.2) the plastic viscosity should tend to the high-shear rate viscosity of blood which is 3.5–4 times greater than that of plasma. In this way, the latter rheological property is allowed to be affected by this impact in a different manner. We can define the relaxation time of blood, χ, as the ratio of the plastic viscosity to the shear modulus:

(13) $$\chi \left(\lambda \right)=\chi_{0}\lambda^{m_{1}}$$

where $\chi_{0}=\;\eta_{0}/G$.

## 3. Rheological Data Fitting and Calculation of the Model Parameters

This section is dedicated to the fitting of our model to experimental data from rheometric flows. The coupled appearance of several elastic and plastic phenomena and the underlying mechanisms and internal variables in TEVP materials, such as blood, necessitates the introduction of a large number of adjustable parameters to describe their rheology. The available experimental data in the literature are limited to those reported in the works of McMillan et al. [47], Armstrong et al. [48] and Bureau et al. [46], which were restricted to a small range of hemodynamical properties eliminating the opportunity of making a parametric correlation between the model’s features and fundamental macroscopic properties such as fibrinogen or hematocrit. Primarily, we use the experiments conducted by McMillan et al. [47] for a healthy subject of systemic hematocrit equal to 45%. The steady-state experiment refers to simple shear flow data providing the shear stress response as a function of the imposed shear rate. Transient experiments are flow curves exported from transient repetitive shearing steps, also known as intermittent steps or rectangular shear steps, with an imposed shear rate equal to $7\;s^{-1}$ with shearing periods lasting 2.5 s, while the intermittent non-shearing intervals have a duration of about 1.5 s. Additionally, we provide the fitting of our model to another subject, and especially we use the steady simple shear, the triangular shear-step and the oscillatory shear data of Donor 1 of the experiments conducted by Armstrong et al. [48]. To extract realistic parameters for the constitutive model, we adopt a non-linear regression [69] on experimental data for steady-state and transient experiments. 

**Figure 1:**
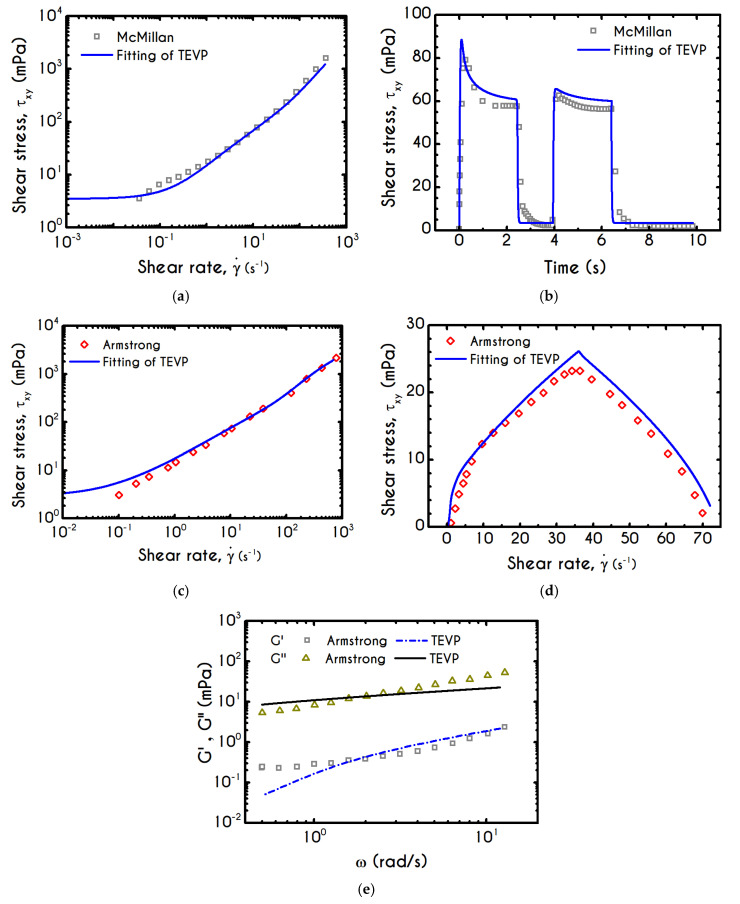
(**a**) Steady flow curve for a simple shear experiment, and (**b**) transient flow curve for an intermittent shear-step experiment. The symbols represent the experimental data for the blood samples reported in [47], and the solid lines represent the nonlinear regression of the TEVP model. (**c**) Steady flow curve of simple shear experiment, (**d**) transient flow curve for a triangular shear-step experiment and (**e**) frequency sweep oscillatory shear experiment. The symbols represent the experimental data of Donor 1 reported in [48], and the solid lines represent the nonlinear regression of the TEVP model.

The fitted curves illustrated in Figure 1, depict an excellent capturing of the major characteristics of both steady shear and transient experiments for both blood samples reported in [47,48], while the values of the adjustable parameters are given in Table 1 for each subject separately. 

The extracted parameters of our model for both subjects are in agreement with the theoretical predictions and the experimental measurements given in [21,72]. Also, both sets of parameters have the same order of magnitude even though the corresponding subjects have different hematological and aging features. We have to point out that shearing in Equation (8) has a dual role, which simultaneously contributes to the dissolution and reconnection of the structural clusters (linear rouleaux and three-dimensional networks). However, its prominent role is to collapse the microstructure necessitating that $k_{3}$ is significantly larger than $k_{2}$. The same pattern is valid for the corresponding exponents where $n_{1}$ is expected to be lower than $n_{2}$ as well as they must be positively defined including $n_{3}$. Another significant issue regarding the fitting of a model is the impact a small change of the input (adjustable parameters) to the output (stresses or microstructure parameter). To make appropriate estimations of the uncertainty of the model’s parameters, the exact values of the experimental errors should also be provided along with the experimental data. None of the known experimental works [46,47,48] gives these errors, and hence we are not able to provide the admissible ranges of our model’s parameters accurately. 

Steady flow properties in simple shear are characterized by the stationary relationship $\tau_{xy}=\tau_{xy}\left(\dot{\gamma }\right),$ which can be obtained in both stress-controlled and shear-rate-controlled experiments. McMillan et al. [47] adopted a rate-controlled technique, which enforces an immediate breakdown of the microstructure and consequently blood begins to fluidize when $\dot{\gamma }>0$. The imposition of a shear-rate that enforces the system to reach a steady state, indicates that stress always exceeds yield stress and the blood is yielded. On the contrary, a flow curve from a stress-controlled rheometer gives a definite interpretation of both yield stress and unyielded region. This means that when applying a stress value, the blood is initially below the yielding point, and thus it is in its solid regime where its behavior is basically elastic, dictating a linear relationship between stress and strain (τ=Gγ). The value above which a clear non-linear behavior between stress and shear rate is observed, indicates the yield stress, $\tau_{y}$. The convexity in the experimental data of McMillan et al. [47] is originated from the experimental protocol, and our fit is in accordance with other similar fittings in [73]. The correct way to obtain the real shear stress – shear rate relationship is to subject the material at a shearing where the shear rate increases initially up to a specific value, and then it decreases to zero. The ‘ramp-down’ section of the flow curve should be used to determine the yield stress of the fluid. Regarding the steady-state shear experiment, the fitting of our model depicts an excellent agreement across the whole range of the shear rates while the predicted yield stress is equal to $\tau_{y}=0.00351\;\mathrm{Pa}$. 

The blood sample is characterized by a hematocrit equal to 45%, but the fibrinogen value is not provided by McMillan et al. [47]. As we have already mentioned, blood rheology is significantly affected by the fibrinogen concentration [25] and consequently it is directly influencing the yield stress magnitude. Morris et al. [74] have proposed a parametric estimation of blood yield stress based on their experimental data. The experiments covered a wide range of fibrinogen concentrations $c_{f}$ (0.1 g/dL–0.9 g/dL) and hematocrits (40–80%). The empirical expression extracted by a fitting procedure is then given by:

(14) $$\tau_{y,c}={\left(-0.091+0.47\;H_{c}+0.22\;c_{f}-0.14\;c_{f}{}^{2}+0.48\;H_{c}\;c_{f}\right)}^{2}$$

where $\tau_{y,c}$ is the yield stress in $\mathrm{dyn}/{\mathrm{cm}}^{2}$ and $c_{f}$ is the fibrinogen concentration in g/dL. Since, the blood sample data used from the current work indicates a yield-stress equal to $\tau_{y}=0.00351\;\mathrm{Pa}$, it corresponds to a fibrinogen concentration equal to 0.1617 g/dL. This is an absolutely physiological value consistent with hemorheological characteristics of the blood samples examined by McMillan et al. [47].

**Figure 2:**
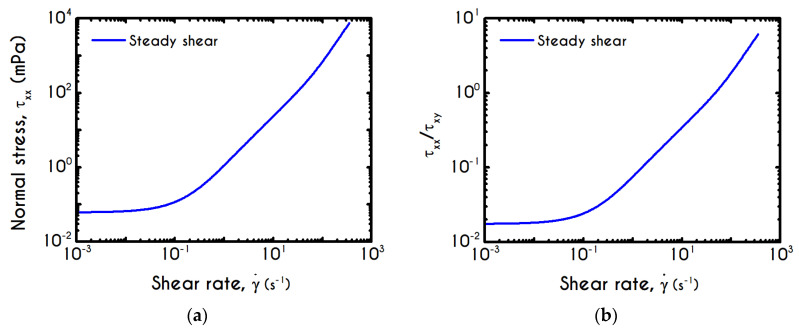
Predictions of our model for (**a**) the normal stress component ($\tau_{xx}$), and (**b**) the ratio $\tau_{xx}/\tau_{xy}$ in steady, simple shear flow, using the set of parameters of Table 1, which refers to the experimental data of blood reported in [47].

Having determined the model parameters from available experiments, we proceed with predictions for other variables under the same two experimental procedures. In Figure 2a,b, we present the predictions of our model for the normal stress component as well as the ratio $\tau_{xx}/\tau_{xy}$ using the rheological parameters of Table 1 exported by the previous fitting procedure. Regarding Figure 2, both normal stress $\tau_{xx}$ and the ratio $\tau_{xx}/\tau_{xy}\;$depict a similar dependence on shear rate. Except for the shear stress, viscoelastic materials or biological systems develop normal stresses even in pure shear flows, which sometimes are found to be equal or to exceed $\tau_{xy}$ [75]. At low values of the imposed shear-rate, our model predicts an almost zero normal stress, while at moderate to high shear rates, the flow curve demonstrates an abrupt increase, especially when $\dot{\gamma }$ exceeds the value of $0.1\;s^{-1}$. From Figure 2b we can observe that normal stress has a significant contribution, and it is comparable to the shear component. For shear rates higher than $100\;s^{-1}$ the ratio $\tau_{xx}/\tau_{xy}\;$is greater than unity and hence $\tau_{xx}$ surpasses the value of $\tau_{xy}$. Apparently, a condition where $\tau_{xx}$ exceeds the $\tau_{xy}$ is not expected for the blood unless the imposed shear-rates are high enough to produce significant velocity gradients in the xx- direction. The xx- stress component is originated at the extension of the plasmatic proteins that bridge the rouleaux components in the x-direction as blood is sheared in the same direction, as well as the extension of the membrane of each RBC [76]. Most of the blood constitutive modelling investigations do not present the normal stress prediction, and hence we are not able to make a comparison with other findings. Varchanis et al. [62] in their work reported a significant contribution of normal stress in simple shear tests and compared their findings with those predicted by the ML-IKH model, which was found to have similar behavior. The presence of normal stresses is suspensions is attributed the intense interaction between the particles. Moreover, the viscoelastic contribution of the plasma due to protein stretching. Similar arguments are presented by Mall-Gleissle et al. [77] for suspensions with viscoelastic matrix fluids.

**Figure 3:**
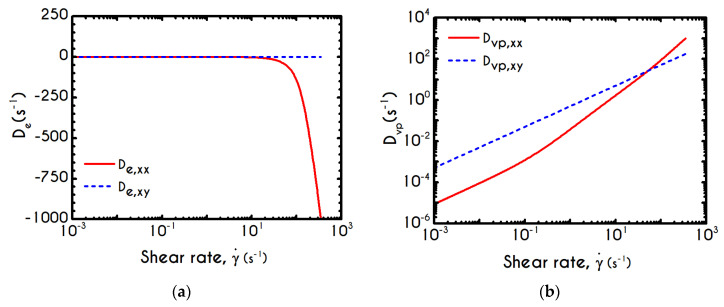
(**a**) Elastic $D_{e,xx}$ and $D_{e,xy}$ and (**b**) viscoplastic $D_{vp,xx}$ & $D_{vp,xy}$, contributions in the deformation rate tensor (Equation (3)) as a function of the imposed shear-rate for the steady, simple shear experiment, using the set of parameters of Table 1, which refers to the experimental data of blood reported in [47].

To further analyze the predicted response, and thus improve the understanding of the underlying mechanisms in terms of macroscopic modeling, we present the separate contributions in the deformation-rate tensor (Equation (3)) using the set of parameters of Table 1 for the experimental data reported in [47]. In particular, the use of the current thixotropic elasto-viscoplastic model, allows us to probe further the shear-rate development in terms of its elastic and viscoplastic contributions. Particularly, Figure 3 illustrates the xx- and xy- components of elastic (Figure 3a) and viscous (Figure 3b) projections as a function of the imposed shear-rate, referring to the steady-state values of the startup test. As we can see in Figure 3, in a steady shear experiment, the developed rate of strain is contributed both by elastic and viscoplastic mechanisms. However, the elastic contribution originates from the xx- component, while the gradients in xy- direction are exactly zero across the whole range of the imposed conditions; this happens only upon steady state ($D_{e,xy}$ is non-zero during the development of flow), is attributed to the fact that the model predicts $\tau_{yy}=0$, and is a standard feature of most constitutive models that induce elastic effects via the upper convected derivative). Above the low shear-rate plateau ($\dot{\gamma }<10\;s^{-1}$), our model predicts an abrupt increase in the absolute value of $D_{e,xx}$, which indicates that blood rouleaux undergoes high strains in the xx- direction. All these agree with our conclusions for the dependence of the stress components on the shear-rate. Figure 3a demonstrates that viscoplasticity is equally significant in both directions for shear-rates up to ~$50\;s^{-1}$. When $\dot{\gamma }$ is higher than $50\;s^{-1}$ the velocity gradients in the xx- direction are high enough to make the contribution of the xx- component of viscoplastic deformation tensor is more significant than the xy- one. Indicatively, for an imposed shear rate ${\dot{\gamma }}_{0}$ equal to $350\;s^{-1}$ the $D_{vp,xx}$ is equal to $988\;s^{-1}$ while the $D_{vp,xy}$ is only $175\;s^{-1}$.

**Figure 4:**
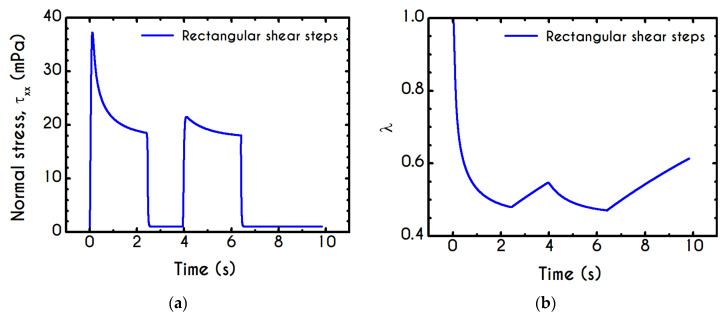
Predictions of our model for (**a**) the normal component of stress $\tau_{xx}$, and (**b**) the structure parameter λ. in transient intermittent shear steps, using the set of parameters of Table 1, which refers to the experimental data of blood reported in [47].

**Figure 5:**
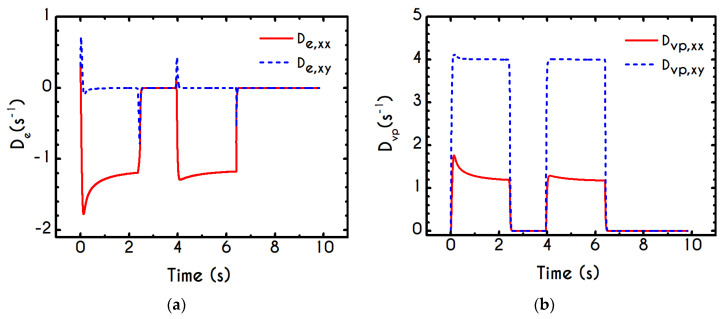
(**a**) Elastic $D_{e,xx}$ and $D_{e,xy}$. and (**b**) viscoplastic $D_{vp,xx}$. and $D_{vp,xy}$ projections of the deformation rate tensor as a function of time for intermittent steps in a shear experiment, using the set of parameters of Table 1, which refers to the experimental data of blood reported in [47].

Using the set of rheological parameters of Table 1 for the experimental data reported in [47], we can predict the normal stress (Figure 4a) and the structure parameter (Figure 4b) for the intermittent shear-step experiment [47]. Quantitively, the evolution of $\tau_{xx}$ resembles that of the shear-stress (Figure 1b). However, similar to the steady experiments, the xx- component takes smaller values than the xy- component (almost half) due to the relatively small value of shear-rate pulses ($7\;s^{-1}$). Carefully studying Figure 4a, we observe an initial overshoot that is a direct consequence of the combination of elastic effects and the thixotropic evolution of the viscosity [1], which is a typical behavior for VE materials or biological systems. We are going to discuss this in detail in the following sections. This can be justified by the implication of the shearing on the stretch of the RBC membrane and the rest of the components of blood, which depicts that the elastic response of blood is significant, especially in the xx- direction, as it is illustrated in Figure 5a. The contribution of the xy- component is negligible, enhancing the fact that stretching of blood is mainly in the flow direction, which may explain the tendency of the RBCs to align with the flow [78]. After the interruption of the shearing, the stresses are drastically reduced until the imposition of the new shearing, producing a second but much milder overshoot. Regarding the structure parameter, it follows a decrease with increasing the shear-rate, indicating a continuous deconstruction of the rouleaux (Figure 4b). This behavior is also observed during the second shear imposition, but the deconstruction of blood follows a much smaller reduction. During the period of flow cessation, the stresses present in the system are small enough to promote the rebuilding of the aggregates, providing a linear increase of λ, which lasts as long as the intermittent steps. Particularly, during the intermittent steps test, the first overshoot is observed at 38 mPa while the second at 21 mPa while the corresponding values for the shear stress are equal to 88 mPa and 65 mPa, respectively. The impact of this test on the structure parameter is high enough to induce a λ = 0.49 at the end of the first shearing while blood manages to recover its microstructure by 15%. After imposing the second shearing, the microstructure is disintegrated again to a value equal to λ = 0.48 while after the cessation of the shearing this value is increased to λ = 0.61 which corresponds to the last observable value in our test. Longer duration of cessation would lead *λ* to increase further.

If we examine the normal elastic component of the deformation-rate (Figure 5a), we will realize that it follows a similar evolution with that predicted for the normal stress (Figure 4a), exhibiting an overshoot at the beginning of each shearing pulse. On the other hand, the shear elastic contribution is zero during the shearing, which means that viscoplasticity dominates the shear forces. Regarding the viscoplastic part of the deformation-rate tensor (Figure 5b), both its components exhibit smoother variations in comparison with the elastic components. In particular, the shear viscoplastic deformation-rate increases smoothly up to $17\;s^{-1}$ at the end of the first pulse, and up to $12\;s^{-1}$ at the end of the second pulse, while the normal component takes an order of magnitude smaller values. 

**Figure 6:**
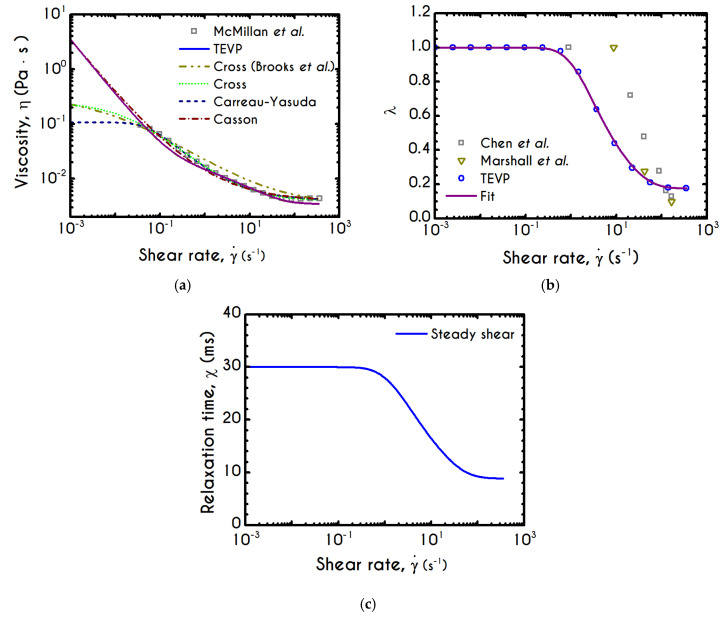
(**a**) Experimental data of McMillan et al. [47] for apparent viscosity along with the nonlinear regression of TEVP, Cross, Carreau-Yasuda, and Casson models. We also provide the predictions of Cross model from the fitting procedure against experimental data of Brooks et al. [79] for the same rheological conditions (Equation (A2)), (**b**) Comparison between the predictions of TEVP model for the structure parameter along with its fitting (Equation (15)) with the modified experimental data reported in [80] and numerical data by [81]. (**c**) Prediction of the relaxation time, χ, as function of shear-rate for McMillan et al. [47] blood samples.

Figure 6a presents the experimental measurements of the apparent viscosity with respect to the imposed shear-rate for a hematocrit equal to 45% from the data reported in [79]. Along with our model predictions for the aforementioned hematocrit, we also reproduce those of the Casson, the Cross, and the Carreau-Yasuda models for improved clarity. The parameters of these models have been determined in Appendix A. 

We observe that our model follows closely the predictions of the Casson model, and also captures the experimental data with an excellent agreement for shear-rates above $1{\;s}^{-1}$, but slightly deviates by 18% from the experimental viscosity below this value. The deviation is strongly associated with the aggregability of blood or in terms of modeling, with the kinetic constants of Equation (8). Regarding the inelastic models, they present an excellent agreement with experiments across their range of validity. However, they provide different predictions for the zero-shear-rate viscosity, which sometimes are unrealistic (e.g., finite zero-shear-rate viscosity). The generalized Newtonian models are algebraic, phenomenological, and able to capture only the steady-state viscous or viscoplastic behavior of blood. In contrast, they are inadequate to reproduce transient as well as elastic effects. Regarding our model’s prediction, the curve fitting in Figure 1 is excellent, even at high shear rates. When the fitting curve of stress is translated into an apparent viscosity curve, at high shear rates, the deviation from the experimental data is more pronounced. When the fitting curve of stress is translated into an apparent viscosity curve, at high shear rates, the deviation from the experimental data is more pronounced. The high-shear experimental viscosity is about $4.22\times 10^{-3}\;\mathrm{Pa}\cdot s$, while the theoretically predicted value is about $3.51\times 10^{-3}\;\mathrm{Pa}\;\cdot s$, which is about 3.5–4 times greater than that of plasma ($1.2\times 10^{-3}\;\mathrm{Pa}\cdot s$). If we bear in mind that: (a) our model is fitted in steady and transient experimental data simultaneously, (b) there are significant experimental errors because at high shear-rates the flow in a cone-and-plate rheometer is inhomogeneous. Our model does not only account for steady-state phenomena but has also been configured to reproduce transient experiments, revealing the complex rheological behavior of blood such as elasto-visco-plasticity along with potential thixotropic effects.

As noted in the introduction, RBC aggregability is an important determinant of hemodynamics [82], particularly due to its association with microcirculatory disorders, mainly cardiovascular diseases [83,84], because the growth of the size of agglomerates may lead to reduced tissue perfusion for example. Although our model does not explicitly account for the RBCs aggregation size, it describes in detail the level of structure of the RBCs, which depends dynamically on the imposed rheological conditions. In Figure 6b, we can see the structure parameter predicted by our model as a function of the shear-rate. For low values of $\dot{\gamma }$, plasticity dominates the flow, the applied stresses are slightly above the yield stress and hence deconstruction term is close to zero. Consequently, blood continues to be in a fully structured state until the shear rate reaches a critical value above which the clustering is starting to collapse. The abrupt reduction of λ is then followed by a stabilized condition where the internal microstructure does not exhibit further deconstruction. Irrespective of the intensity of shear rate, λ never reaches 0, but asymptotes to a finite value indicating that the material never becomes fully unstructured. In order to make a sufficient comparison of our model predictions with those exported by experimental observations of the rouleau size, we invoke the work of Chen et al. [80]. They provided quantitative measures of RBC aggregability from direct visual monitoring of the aggregation and disaggregation processes. If we consider that at the lowest shear rate, the level of structure is the highest and equal to unity, we are able to correlate any other state of the blood as a percentage of the fully structured state. Before comparison, we transform the median aggregate size of RBCs given by Chen et al. by dividing these values with the average size at the zero-shear rate. After that, Figure 6b illustrates the predictions of TEVP along with the transformed data of RBC median aggregate size at equilibrium as a function of shear rate. Qualitatively the model predictions are in quite good agreement with the experimental data if one considers that our simulation accounts for a hematocrit value equal to 45%. In comparison, the experiment has been conducted for a significantly lower value of about 10%. As a result, in our case, the interaction of the dissolved phase is more intense and “unjamming” and “disentanglment” phenomena appear at lower values of the applied shear rate, something that is consistent with the data that we present. Despite the large deviation on the hematocrit level, our model captures the continuous deconstruction of blood’s microstructure as shear rate increases but with a steeper way than the experimental observation. Although the quantitative deviation is great enough below $80\;s^{-1}$, for higher values of the shear rate, we observe an overall discrepancy of about 12%. It should be mentioned that the average aggregation size can be optically measured in quite transparent systems when the suspension or the blood is relatively dilute (e.g., systemic hematocrit below 15%). For the sake of convenience, we also illustrate the fit of a sigmoid like function ($\lambda_{s}\left(\dot{\gamma }\right)$) on our theoretical predictions of the TEVP model in order to provide a more natural way to reproduce the predicted microstructural response. The proposed function of the thixotropic structure parameter $\lambda_{s}$ distribution with respect to shear rate is given by the following expression, while the corresponding parameters are illustrated in Table 2:

(15) $$\lambda_{s}\left(\dot{\gamma }\right)\;=\;A\;+\;\left(B-A\right)\frac{{\dot{\gamma }}^{n}}{\left(k^{n}+{\dot{\gamma }}^{n}\right)}$$

The relaxation time of the blood sample, χ, is given by Equations (13) and (15). It exhibits a similar sigmoidal variation as a function of the shear-rate $\dot{\gamma }$ (Figure 6c). It smoothly decays from $\chi_{0}$ to 9.1 ms, which is the asymptotic value in the limit of high shear-rates. The variation occurs in the range of $1\;s^{-1}$ to $90\;s^{-1}$, where rouleaux rearrangements and disintegration take place [85]. Although we have a qualitative agreement with the experiments by Thurston & Henderson [85] for the range of variation of the relaxation time, there is a deviation between the model predictions and their estimations. This is strongly related to the experimental procedure and the theoretical approximation that Thurston & Henderson [85] have adopted. Specifically, they provide estimates of the relaxation-time based on the definitions of the theory of linear viscoelasticity (Maxwell model, which is valid only for infinitesimal deformations), while the experiments where performed in the nonlinear regime (Bird et al. [86]). They made this poor assumption in the whole range of shear-rate. Thus, from this work, we can only be sure for the shear-thinning dependence of the relaxation time, which match pretty good with the prediction of TEVP model. However, there is no indication that experiments were done in the limit of infinitesimal strains. On the contrary, the imposition of any finite rate of strain (or velocity field) causes the yielding of the blood sample and its flow. Thus, the experiments were executed in the nonlinear regime, where the Maxwell theory is not valid. However, the systematic misassumption that they introduced in their calculation, does not affect the dependence of the relaxation time on the shear rate, but only affects the magnitude of the blood relaxation time. 

## 4. Steady and Transient Rheological Predictions

In the current section, we provide a detailed description of the rheological predictions of the model regarding the thixotropic elasto-visco-plastic rheological behavior of blood in standard rheometric flow experiments for the set of the parameters reported in Table 1, which refers to the experimental data of blood reported in [47]. There are simple flows that can easily ascertain the nature of blood and more complex flow kinematics where different rheological aspects of blood can coexist, especially when the time scales of these phenomena are comparable. Taking into consideration the limited available data in the literature, we offer an extended set of predictions in distinct flow kinematics. This is an imperative method to ensure that our model is able to produce a sensible rheological behavior of blood, even in flows that have not been tested yet. We include predictions of startup, startup followed by cessation, intermittent steps, as well as large amplitude oscillatory shear (LAOS) flows. The general shear flow kinematics is then given by:

(16) $$\begin{array}{ccc} \nabla u=\left(\begin{array}{cc} 0 & 0\\ \dot{\gamma } & 0 \end{array}\right) & , & D=\frac{1}{2}\left(\begin{array}{cc} 0 & \dot{\gamma }\\ \dot{\gamma } & 0 \end{array}\right) \end{array}$$

At any kind of experiment, steady or transient, the flow field is assumed to be known a priori and given by Equation (16). The numerical method that we use for the solution of the system is a second-order, predictor-corrector implicit scheme with an adjustable time step. The reduced form of the Equations (3)–(6) for each flow kinematics is presented in detail in Appendix B.

### 4.1. Startup

The so-called startup experiment is the first and simplest test used for the evaluation of new models. It provides some very useful information for the dynamics and the structural evolution of blood. The kinematics of this flow imposes that the blood is subjected to a step-change in the applied shear-rate until the steady-state is satisfied. Here, $\dot{\gamma }$ acquires immediately the prespecified value ${\dot{\gamma }}_{0}$. The flow kinematics for the startup shear experiment is given by:

(17) $$\nabla u=\left(\begin{array}{cc} 0 & 0\\ {\dot{\gamma }}_{0} & 0 \end{array}\right)\;\;,\;\;t\geq 0\;$$

**Figure 7:**
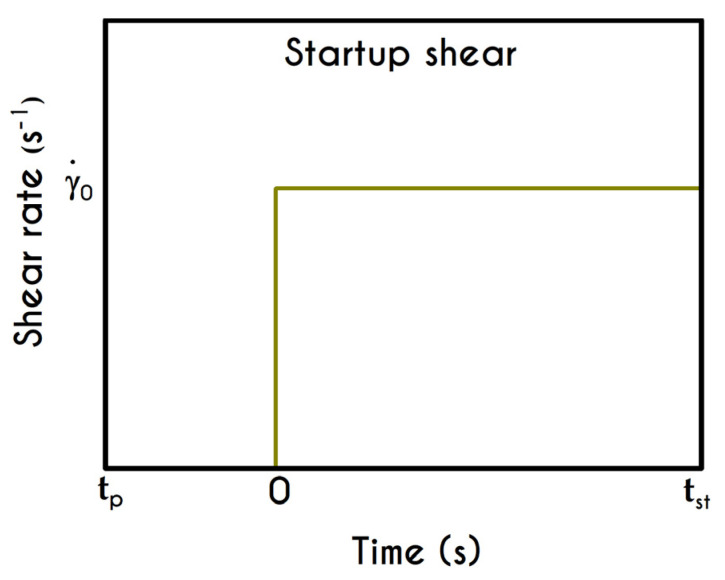
Startup shear experiment.

The graphic representation of the aforementioned form of the shear experiment is illustrated in Figure 7.

**Figure 8:**
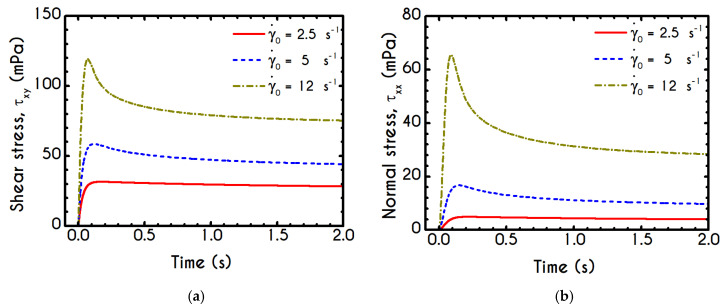
Predictions of our model for the time evolution of the (**a**) shear and (**b**) normal components of the stress, for values of ${\dot{\gamma }}_{0}$ equal to $2.5\;s^{-1}$, $5{\;s}^{-1}$, and $12{\;s}^{-1}$.

In Figure 8, we illustrate the stress evolution, both shear and normal, for the startup experiment. The imposed shear rate values of ${\dot{\gamma }}_{0}$ are equal to $2.5{\;s}^{-1}$, $5{\;s}^{-1}$, and $12{\;s}^{-1}$. It is important to note that the imposition of a deformation rate, instead of a stress, would always lead to the blood yielding. In other words, at steady state, all cases are satisfying the von Mises criterion, and the blood sample begins to fluidize after the surpassing of the yield stress. After the imposition of a shear rate, the stresses are gradually increasing, and the shear stress attains a maximum, which arises at different time instances depending on the applied shear rate ${\dot{\gamma }}_{0}$ (Figure 8a). The initial rise in the shear-stress is caused by blood elasticity, as already mentioned in the previous section. The contribution of the two forms of the erythrocytes is directly affected by the shear stress presented in the system as well as the duration of the shearing. The dynamic equilibrium shifts toward more individual cells when the applied shear rate is increased, and the blood behaves as an elastic solid when the resulting shear stress is below its yield stress. So, this maximum in stress denotes that the dynamic energy stored in the form of elasticity is slowly released due to the destruction of the aggregates, driving the dissipative motion of individual RBCs and smaller rouleaux. The larger the value of the imposed shear-rate, the earlier the formation of the overshoot due to enhancement of the breakdown mechanism of the rouleaux [5,87] or the stronger effect of Equations (10) and (11) in the evolution of the structure parameter. Thus, we observe an initial elastic development of the stress, followed by a thixotropic relaxation, which is typical behavior of blood as it is confirmed experimentally [40]. The elastic property is mainly caused by stretching the rouleaux network, which tends to collapse when the applied shear stress is higher than the yield stress, and the blood is free to flow like a liquid with shear-thinning characteristics.

It is also observed that for low shear-rates, the stress relaxation is not very intense, the overshoot is almost absent, and the blood sample achieves a steady-state faster. This is clearly originated from the different time scalings of elasticity and thixotropy, which in the case of low shear rate is not so intense, the formation and breakdown processes are similarly milder, and the microstructural rearrangements are more limited. For higher imposed shear rates, the resistance to the change of the state of the blood is more intense and much faster, while the destruction of the microstructure and subsequent relaxation follow faster dynamics. Thus, the overshoot region is narrower, and the developed stresses are higher than in lower shear rates. The same pattern is followed by the xx- component of the stress tensor, as illustrated in Figure 8b. We observe a qualitative similarity between normal and shear stress predictions, but quantitatively, the results for the xy– component are one order of magnitude higher than that of the xx– counterpart. A qualitative difference for ${\dot{\gamma }}_{0}=12\;s^{-1}$, which depicts a higher deviation from ${\dot{\gamma }}_{0}=5\;s^{-1}$ predictions for the case of the xx- component is also observed. Although in some cases the prediction of normal stress could be significant, our model does not predict a considerable contribution of $\tau_{xx}$ compared to $\tau_{xy}$. The relevance between the two components of the stress tensor can be elucidated by the fraction of $\tau_{xx}/\tau_{xy}$. For the given cases, the normal component is always more than an order of magnitude smaller than $\tau_{xy}$.

**Figure 9:**
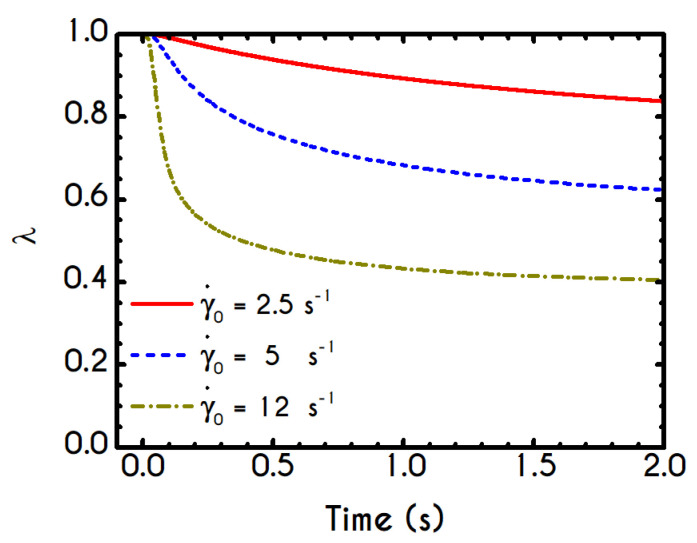
Predictions of our model for the time evolution of the structure parameter λ in startup shear flow, for values of ${\dot{\gamma }}_{0}$ equal to $2.5\;s^{-1}$, $5{\;s}^{-1}$, and $12{\;s}^{-1}$.

A detailed picture of the structure evolution in the fluid in terms of λ and its dependence on the imposed shear-rate can be seen in Figure 9. The blood possesses a yield stress due to electrostatic forces between plasma proteins and neighboring RBCs, and at startup, it assumes macroscopically a hyperelastic, neo-Hookean behavior, while $D_{vp}=0$. Thus, blood is fully structured (λ = 1) at any time before yielding $t\;<\;t_{yielding}$, until the developed stresses satisfy the von Mises criterion, and the blood begins to fluidize. Blood needs to sustain lower stresses to support the fluidization process; thus, a thixotropic stress relaxation is observed. Beginning with λ(t=0) = 1, there are three distinct responses of the blood, which are becoming clearer as ${\dot{\gamma }}_{0}\;$attains higher values. Initially, the von Mises criterion is not satisfied, and the structure parameter does not change, because φ in Equation (11) is equal to zero. Next, the transition to the liquid state begins and λ decreases, as the magnitude of the effective stress increases over time. As expected, the applied shear-rate affects the thixotropic response of blood. For higher shear-rates, the destruction of the microstructure is quicker and more intense, and the structure variable decreases earlier and more abruptly, but qualitatively all the curves have similar behavior. Irrespective of the intensity of shearing, λ never reaches 0, but asymptotes to zero for extreme values of the shear rate. In other words, blood never becomes fully unstructured., which agrees with the experimental data by Karabetsos et al. [88]. The relaxation time follows almost the same pattern as the structure parameter (Equation (13)), exhibiting a smoother variation. All relaxation-time curves start from $\chi_{0}$ and end-up with values illustrated in Figure 6c depending on the applied shear-rate. 

**Figure 10:**
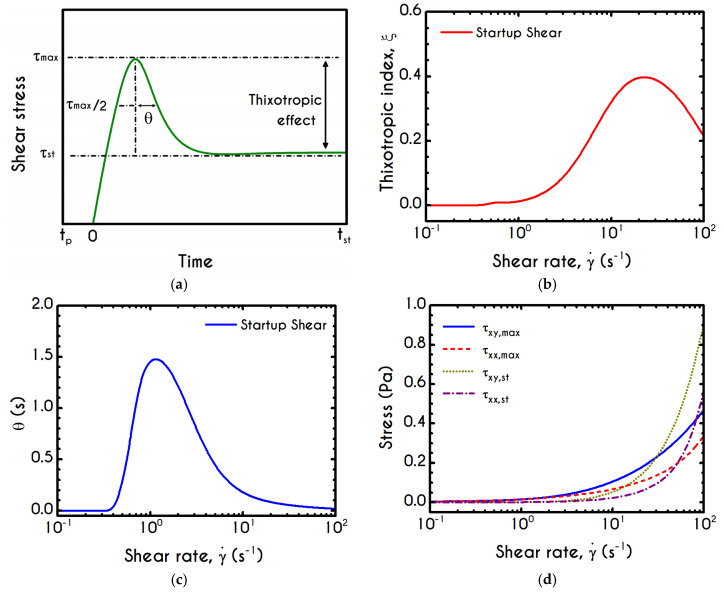
(**a**) Definition of the thixotropic index in startup shear experiment, (**b**) Thixotropic index as a function of shear rate in startup experiment, (**c**) Prediction of quantity θ with respect to imposed shear rate in startup experiment, and (**d**) Maximum observable value (overshoot) and steady state values of $\tau_{xx}$ and $\tau_{xy}$ as a function of shear rate for startup experiment.

For both normal and shear components of the stress tensor, the time evolution of the experiment is quantified by using a dimensionless ratio, called thixotropic index ξ [87]. The latter is associated with the intensity of the thixotropic effect by comparing the observable overshoots with the time at which steady-state is achieved, namely the mechanism of the elasto-thixotropic response of blood. The thixotropic index is defined as:

(18) $$\xi \left(\dot{\gamma }\right)=\;\frac{\tau_{max}-\tau_{st}}{\tau_{st}\;\theta \;\;\dot{\gamma }}\;$$

where $\tau_{max}$ and $\tau_{st}$ are respectively the maximal and the stationary values of the shear stress and θ is the time required for the shear stress to decrease from the maximum value to the half peak value $\left(\tau_{max}-\tau_{st}\right)/2$. All the aforementioned quantities are graphically represented in Figure 10a. Apparently, the rheological response of blood and, consequently, the thixotropic index can be directly correlated to the internal structure of the RBCs clustering and hence the rouleaux configuration. This network is progressively “disentangled” by shear stresses until an equilibrium state between the network of rouleaux and the individual cells is reached. Except for the rate of deformation, which considerably affects the thixotropic effect of blood in startup experiments, a significant contribution comes from the alteration of proteinic factors that promote the bridging of the adjacent cells and hence the development of the agglomeration. Consequently, with an increase in fibrinogen concentration, the initial response of the blood due to elastic forces is stronger, the overshoot size is greater, and the steady stress value is sufficiently smaller, yielding an increase in ξ [9]. As argued by Lacombe et al. [9], the higher the thixotropic index, the more intense is the RBC disaggregability. This assertion is explained by the fact that elastic effects are less pronounced in blood samples where the formation of the rouleaux is weaker due to low binding forces.

Since our investigation is restricted to a single set of hematocrit and fibrinogen values, the only factor that may affect the thixotropic index is the shear-rate magnitude, $\;\dot{\gamma }$. Figure 10b values the thixotropic index as a function of the imposed shear-rate for the startup experiment. Above the low shear-rate plateau at $\dot{\gamma }=1{\;s}^{-1}$, the thixotropic index follows a fast increment with the increase in shear-rate up to a critical value. The maximum of the thixotropic index is reached at $\dot{\gamma }=\;23.71{\;s}^{-1}\;$and equals 0.40. Within this range of shear-rate, the elastic response, and consequently, the maximum obtainable stress is large, while the transition from pick to steady state is quickly achieved. This can be justified by the θ parameter prediction as a function of shear-rate in Figure 10c, which demonstrates a continuous decrease of θ after a short-term increment, which is limited to the initial low values. Particularly, θ has a zero value up to $\dot{\gamma }=0.42\;{\;s}^{-1}$ after which it exhibits a steep increment to a critical value where it shows a maximum value at $\dot{\gamma }=1\;{\;s}^{-1}$ of about θ=1.5 s. Further increase of shear rate does not affect the θ time promoting a steady-state condition, which in turn causes a significant reduction on the thixotropic index. This reduction exclusively originates from unchanged θ since the maximum stress present in the system is continuously increasing.

### 4.2. Effect of Variation of Shear Rate with Time

**Figure 11:**
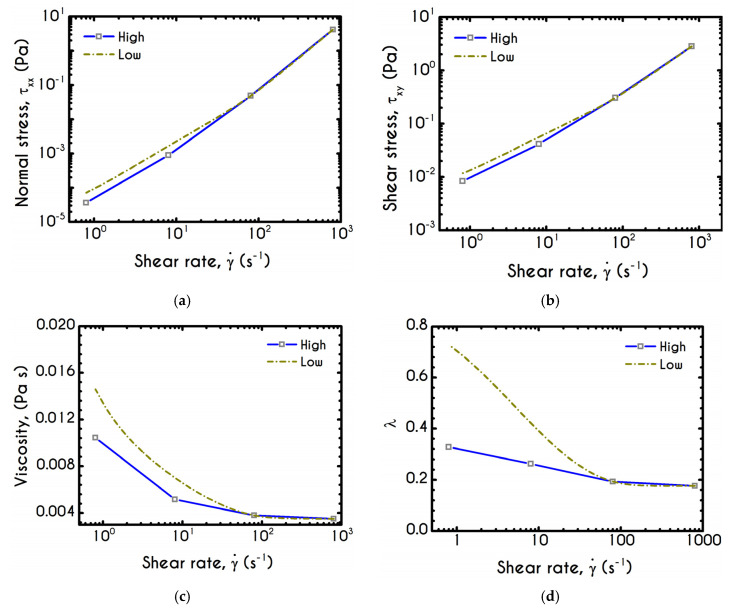
The effect of low and high $\ddot{\gamma }$ on (**a**) the normal stress$\;\tau_{xx}$, (**b**) the shear stress $\tau_{xy}$, (**c**) the apparent shear viscosity, and (**d**) the structure parameter λ.

An important issue that commonly arises in experimental hemorheology is the effect of the variation of the shear-rate with time on the stress development and microstructural response as well as the accuracy in the measurements of the steady shear viscosity (Figure 6a). This kind of test has been previously done by Kaliviotis and Yianneskis [58]. The test is conducted for two different variations of the shear-rate with time, i.e., for the derivative of shear rate $\ddot{\gamma }$, but for the same range of shear rates. The process starts from a shear rate equal to ${\dot{\gamma }}_{s}=\;800\;s^{-1}$ to ${\dot{\gamma }}_{e}=\;0.8{\;s}^{-1}$ with a low and a high rate of shear rate which is given by:

(19) $$\ddot{\gamma }\left(t\right)=\;-\;\frac{{\dot{\gamma }}_{0}}{\alpha \Delta t}\;e^{\left(\frac{-t}{a\Delta t}\right)}$$

which in turn comes from the definition of the shear rate according to Kaliviotis and Yanneskis [58] as:

(20) $$\dot{\gamma }\left(t\right)=\;{\dot{\gamma }}_{0}\;e^{\left(\frac{-t}{a\Delta t}\right)}$$

where a is the number of steps from ${\dot{\gamma }}_{s}$ to ${\dot{\gamma }}_{e}$, ${\dot{\gamma }}_{0}$ is the imposed shear-rate for each step, t is the time, and Δt is the duration of shearing in each step. The latter quantity is the same for both cases and equal to 1 s, but for the first case we choose a low number of transitional steps which is equal to $a_{low}=4$, while for the case with high variations the corresponding factor is equal to $a_{high}=30$. Obviously, the duration of each simulation is different, but they lie in the same shear rate range. The selected rheological conditions are those proposed by Kaliviotis and Yanneskis [58]. They performed rheometric tests for the evaluation of the apparent shear viscosity in parallel with image analysis for the description of the aggregation size index. Our results demonstrate a reasonable behavior for the stress prediction, the apparent viscosity, and the structure parameter. Figure 11 shows that there is a considerable difference in the measured viscosities as well as in the microstructural state of blood between the examined cases. Particularly, as $\ddot{\gamma }$ decreases, thixotropy emerges, and consequently, RBCs have enough time for the rebuilding process. In case with $a_{high}$, blood is subjected to steep changes in shear rates, and therefore the thixotropic response is absent, the buildup process is weaker and the final state at ${\dot{\gamma }}_{e}$ demonstrates a reduction of apparent shear viscosity compared to that predicted for $a_{low}$ (Figure 11c). Ιt is evident that the way in which the transition between shear rates takes place has a great impact on the configuration of the structure parameter. Figure 11d justifies the aforementioned remarks on how shear rate variations affect blood behavior. Indeed, our model predicts that in the case of $a_{low}$, the structure parameter λ demonstrates a significant deviation from the $a_{high}$ counterpart which is about two times greater. Having in mind that the rheological conditions are the same for these cases, the observed deviation is considerable, confirming that the interplay between thixotropy and how the experiment is performed are strongly dependent.

### 4.3. Shear Cessation

In this test, the blood is being sheared with a prespecified rate of strain ${\dot{\gamma }}_{0}$ followed by a cessation of the flow at a specific instant, and then the material is allowed to settle until a steady-state is achieved. Such an experiment is useful for the evaluation of the buildup mechanism in structural kinetics, and also reveals the thixotropic nature of blood through the evolution of structure parameter λ over the time. In an earlier work, Bureau et al. [89] introduced this kind of experiment to probe the rheological response of normal and pathological human blood to a step-change in shear-rate protocol. They conducted shear cessation testes for two imposed shear rates, namely for $0.05{\;s}^{-1}$ and $1{\;s}^{-1}$. In this section, we examine the blood rheological response in higher values than those proposed by Bureau et al. [89] and specifically for $7.1\;s^{-1}$, and $14.1{\;s}^{-1}$. The initial conditions imposed for the solution of the governing equations are those corresponding to an initially unyielded and unperturbed material. To this end, we assume a zero initialization for the stress field while the structure parameter is assumed to be unity. The moment when the shearing is ceased is the same for all cases and equal to $t_{ces}\;=\;3\;s$ and the experiment is terminated at $t_{st}$ which indicates that a steady state solution is satisfied. The flow kinematics for the shear cessation experiment is given by:

(21) $$\{\begin{array}{c} \nabla u=\left(\begin{array}{cc} 0 & 0\\ {\dot{\gamma }}_{0} & 0 \end{array}\right)\;\;,\;\;t<t_{ces}\\ \nabla u=\left(\begin{array}{cc} 0 & 0\\ 0 & 0 \end{array}\right)\;\;,\;\;t\geq t_{ces} \end{array}\;$$

**Figure 12:**
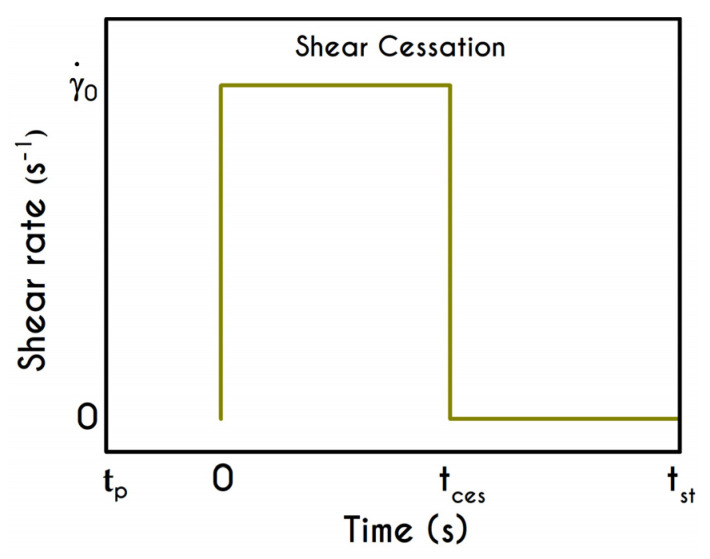
The shear cessation experiment.

**Figure 13:**
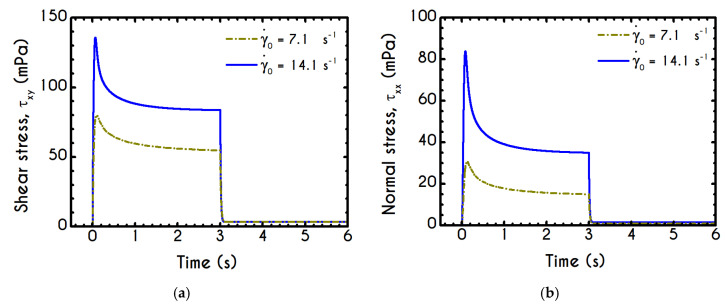
Predictions of our model for the time evolution of (**a**) the shear, and (**b**) the normal components of the stress tensor in shear cessation flow for values of ${\dot{\gamma }}_{0}$ equal to $7.1{\;s}^{-1}$, and $14.1{\;s}^{-1}$.

The graphic representation of the shear cessation experiment is illustrated in Figure 12, while the shear and normal stress evolution during this test are given in Figure 13. The predictions of our model for various shear rates show that upon cessation of the flow, the stress curves have different behavior, depending on the magnitude of the rate of strain. In accordance with the shear flow experiment, an overshoot is formed at the beginning of the shearing imposition originated from the elasto-thixotropic nature of the blood.

As already mentioned in this work, the thixotropic time scale in blood is significantly greater than viscoelastic time scale [33]. This property can result in large overshoot predictions at short times, accompanied by undershoot predictions at larger times. The existence of such undershoots is controversial [90]. In the case of more viscoelastic suspensions, the maximum overshoot appears at longer times, and the probability of predicting an undershoot decreases substantially [91]. As the imposed shear-rate is higher, the shock of the suspension is larger, and consequently, the stored elastic stresses attain higher values. The larger the value of the imposed shear-rate, the earlier is the appearance of the overshoot. In particular, regarding the shear stress (Figure 13a) the observable overshoots are formed at 70 mPa and 136 mPa for ${\dot{\gamma }}_{0}=\;7.1\;s^{-1}$ and ${\dot{\gamma }}_{0}=\;14.1\;s^{-1}$ respectively. The corresponding values for normal stress (Figure 13b) are smaller compared to those predicted for the shear stress and are equal to 30 mPa and 85 Pa for ${\dot{\gamma }}_{0}=\;7.1\;s^{-1}$ and ${\dot{\gamma }}_{0}=\;14.1\;s^{-1}$ respectively. When the steady-state is achieved, the shearing is interrupted, and from this point onward blood is entering a relaxation mode. At the same time, the stresses abruptly decrease to a small but non-zero value which is the yield stress of the blood. Eventually, we observe residual stresses after the cessation of the shearing which are equal to 3.5 mPa for the shear component of both cases. The stress ratio $\tau_{xx}/\tau_{xy}$ always take small values, smaller than 0.4.

**Figure 14:**
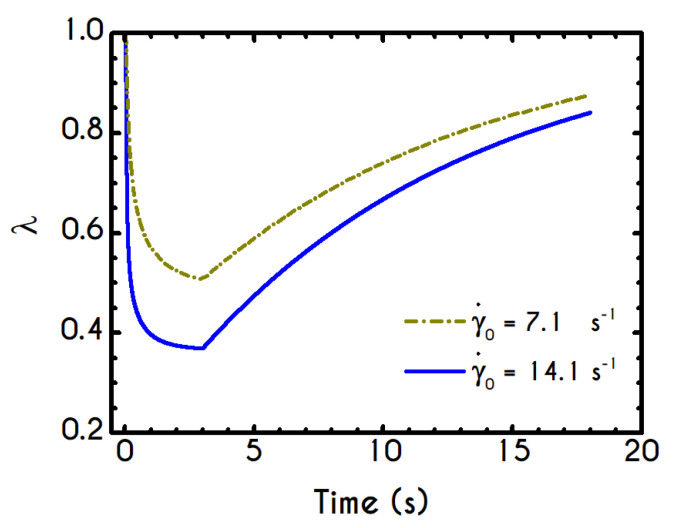
Predictions of our model for the time evolution of the structure parameter λ in shear cessation flow for values of ${\dot{\gamma }}_{0}$ equal to $7.1\;s^{-1}$, and $14.1{\;s}^{-1}$.

In general, the response of the blood is identical to that of the previous experiment, and the only interesting quantity is the evolution of the structure parameter after the interruption of the shearing, depicted in Figure 14. Initially, the blood is unperturbed dictating a λ(t=0)=1, while it is maintained in the same state for some time, which depends on the imposed conditions. The higher the imposed shear-rate, the shorter is the period in which the blood’s state remains unchanged. After this point, the stresses present in the system satisfy the von-Mises criterion of Equation (6), and the microstructural aggregates begin to collapse. The microstructure no longer resists the imposed strain, and it is getting softer with an abrupt decrease in λ. The higher the shear-rate, the more intense and earlier is the reduction of λ, while a low rate of strain allows a more gradual and limited disaggregation of the microstructures up to time $t_{ces}\;=\;3\;s$, when the flow ceases. Particularly, the microstructure parameter experiences a reduction from unity to 0.50 and 0.36 for ${\dot{\gamma }}_{0}=\;7.1\;s^{-1}$, and ${\dot{\gamma }}_{0}=\;14.1\;s^{-1}$ respectively. The cessation of the flow gives rise to the reconstruction process through the build-up term because the magnitude of the effective stress is zero. The rouleaux formation (rejuvenation) is intense and quick as depicted by the abrupt increase of the parameter λ, which demonstrates a linear behavior depending on the imposed shear rate ${\dot{\gamma }}_{0}$. Indicatively, after the cessation of the shearing, the reaggregation of the blood microstructure has a constant increase rate equal to 3.96 % and 5.08 % for ${\dot{\gamma }}_{0}=\;7.1\;s^{-1}$, and ${\dot{\gamma }}_{0}=\;14.1\;s^{-1}$ respectively. Blood is gradually getting harder, which is justified by the rebuilding process in an attempt to restore its original state. 

### 4.4. Variation of Cessation Segment in Rectangular Shear Steps

The rectangular shear-step test, also known as intermittent steps test, is composed of two consecutive shear cessation steps with a varied duration, [59,62]. This is another rheological test that has been reported in various investigations to evaluate the thixotropic response as well as the elastic aspect of the material. Similarly, to the startup test that was previously studied, blood is being subjected to a sequence of shearing followed by cessation of the flow over time. Initially, blood is in a fully structured state, resulting in zero stresses while λ(t=0)=1. The flow kinematics for the rectangular shear steps experiment is given by:

(22) $$\{\begin{array}{c} \nabla u=\left(\begin{array}{cc} 0 & 0\\ 0 & 0 \end{array}\right)\;\;\;\;\;,\;\;\;\;\;\;\;\;\;\;\;\;\;\;\;\;\;\;t_{1}\leq t\leq t_{2}\cup^{\;}\;t\geq t_{3}\\ \;\;\;\nabla u=\left(\begin{array}{cc} 0 & 0\\ {\dot{\gamma }}_{0} & 0 \end{array}\right)\;\;,\;\;\;\;\;\;\;\;\;\;\;0<t<t_{1}\cup^{\;}\;t_{2}<t<t_{3} \end{array}\;$$

**Figure 15:**
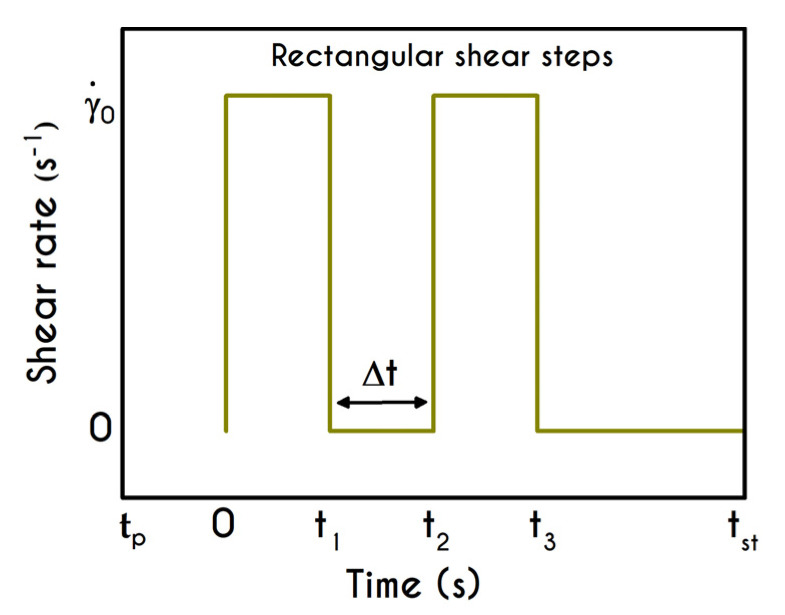
Test input graphical representation for the intermittent steps (or multiple startups and cessations) experiment.

The graphical representation of the intermittent steps experiment is illustrated in Figure 15. This two-step test is characterized by the imposed shear rate ${\dot{\gamma }}_{0}$ as well as a period of time, denoted by Δt, which is the intermediate duration of two repetitive shearing impositions. Δt is the critical factor for this test since it controls the dynamics of rouleaux formation and consequently the maximum values of stresses.

**Figure 16:**
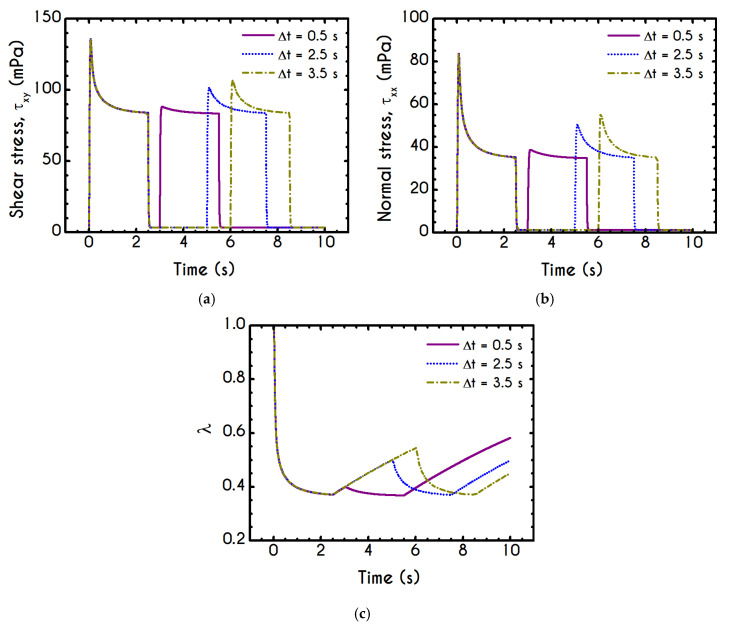
Predictions of our model for the time evolution of (**a**) the shear $\tau_{xy}$, (**b**) the normal $\tau_{xx}$ components of the stress tensor, and (**c**) the structure parameter in intermittent-step test for ${\dot{\gamma }}_{0}=\;14.1\;s^{-1}\;$and Δt equal to 0.5 s, 2.5 s, and 3.5 s.

We conduct three distinct cases having different intermediate periods and specifically for Δt equal to 0.5 s (Case 1), 2.5 s (Case 2), and 3.5 s (Case 3) for a shear rate value equal to ${\dot{\gamma }}_{0}=\;14.1\;s^{-1}$. Figure 16 shows the shear and normal components of stress tensor with respect to time, as well as the evolution of the structure parameter of the examined cases. The predictions of xx– and xy– components of the stress tensor depict a similar trend for all cases. The size of the initial overshoot is essentially configured by the imposed shear rate, which is strongly linked to the elasto-thixotropy of blood. Immediately after yielding, the stored energy is released driving the dissipative flow of the blood, the structure is starting to break down, and the structure parameter causes an effective reduction of the thixotropic viscosity $\eta_{t}$, while the shear stress decays until the first cessation at t =2.5 s. Then, the interruption of the imposed shear rate leads to a quick and abrupt reduction of the developed stress, which attains a new non-zero value. Similarly to the previous test, the residual stress within the sample are equal to 3.5 mPa for the shear component for all cases. In general, the latter is formed due to the inability of the sample to completely relax and return to its original state. The magnitude of the residual stresses does not only depend on the imposed shear rate but also on the duration of the shearing interruption, Δt. Blood is maintained at this state for a time equal to Δt, and again it is subjected to a new step in shear characterized by the same ${\dot{\gamma }}_{0}$. Interestingly, a second overshoot is observed, the size of which dependents on the duration of intermittent steps. Naively, the blood should not behave differently at the second shearing since the stresses that have developed in the material are the same for all cases before it starts. However, the initial state of the blood before the imposition of the second shearing is not the same in all cases due to the rejuvenation process. For the case where Δt is small, such as Case 1, blood does not have enough time to relax completely, and consequently, the second shock is quite milder compared with the previous one. The longer the time between the shear impositions, the higher is the reconstruction of the microstructure of blood and, consequently, the formation of the second overshoot. Apparently, the shock of the second shearing imposition for Case 3 is higher than that of Case 2, which has less time to return to its initial state. The flow curves for xx– and xy– components are qualitatively comparable, but they differ significantly in the order of magnitude. However, if the duration of the cessation is higher than a critical value of $\Delta t_{crit}=1/k_{1}\;,$ the Δt has absolutely no effect on the rheological behavior of blood under the second shearing. Particularly, this critical value is approximately equal to 11 s. Another difference is the formation of the first overshoot which in the case of $\tau_{xx}$ is significantly steeper. Concerning the impact of the intermediate duration of this test on the developed stresses, we observe a reduction in the overshoot size between the two adjacent steps equal to 55% and 42% for Case 1 for xx– and xy– components, respectively. For Case 2, the aforementioned percentages are equal to 38% and 32%, and Case 3 demonstrates a reduction equal to 34% and 28% for xx– and xy– components respectively. 

Figure 16c illustrates the response of the blood structure to intermittent step shear simulation through the structure parameter λ. Obviously, Δt directly affects the thixotropic state of blood through the change of the residual stresses. After the interruption of the shearing, the rouleaux start to reform, and thus, an increase in λ is observed. The longer Δt is, the higher is the thixotropic reconstruction of the rouleaux, which lasts as long as the duration of the cessation step. For higher imposed Δt, the destruction of the microstructure and subsequent relaxation follow faster dynamics. For these cases, the dominant term in the differential equation of λ is that of collapse, and the steady-state is achieved very quickly. The following cessation of the flow gives rise to the rejuvenation process, and the blood starts to build-up the microstructural clustering again. 

### 4.5. Triangular Shear Step

A significant test for the adequate modelling of any TEVP material is the hysteresis (triangle ramp) experiment, in order to probe the viscoelastic and the thixotropic characteristic times simultaneously. It constitutes of a ramp-up followed by a ramp-down in shear-rate. The shape of the hysteresis curve depends sensitively on two experimental parameters (Bureau et al. [46,89]): The rate of change of the shear-rate a (Equation (23)), which is a factor that reflects the rate of increase/decrease in shear rate measured in $s^{-2}$, and the duration of the shearing in each direction, denoted by $t_{max}$. The current section is dedicated to the presentation of the predictions of our TEVP model regarding the triangular step test.

**Figure 17:**
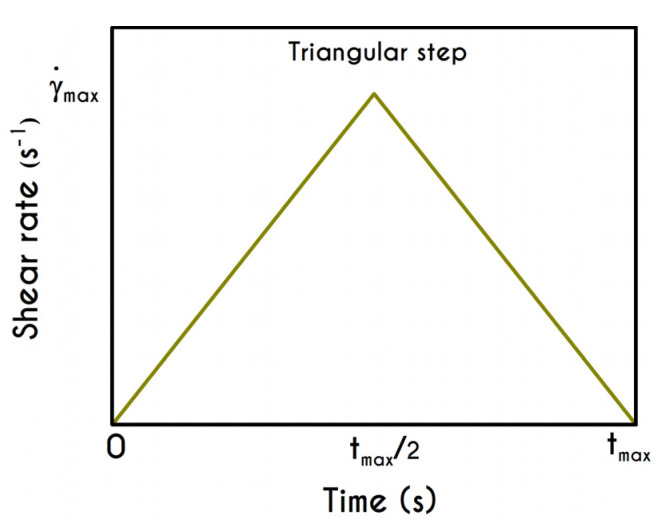
Test input for the triangular step in the shear experiment.

The current analysis encompasses the predictions of six distinct rheological conditions in terms of the a and $t_{max}$. These characteristics were chosen as they can represent typical triangle ramp conditions [48]. In an experiment, the sample meets a wide range of shear rate values, which are low enough that it is still structured, but not so small that there is only an elastic contribution. The flow kinematics for the triangular step experiment is given by the following expression, while the graphical representation is illustrated in Figure 17:

(23) $$\{\begin{array}{c} \nabla u=\left(\begin{array}{cc} 0 & 0\\ \;\;\;a\;t & 0 \end{array}\right)\;\;,\;\;t<t_{max}/2\\ \nabla u=\left(\begin{array}{cc} 0 & 0\\ -a\;t & 0 \end{array}\right)\;\;,\;\;t\geq t_{max}/2 \end{array}\;$$

The required quantities are those of a and $t_{max}$, while ${\dot{\gamma }}_{max}$ arises from the interrelationship of these quantities. The kinematics of the first case (Case 1) is characterized by rates equal to $a=5{\;s}^{-2}$, $a=10\;s^{-2}$, and $a=20\;s^{-2}$ for a maximum duration equal to $t_{max}=\;6\;s$. The second case (Case 2) is characterized by a higher duration equal to $t_{max}=\;15\;s$ accompanied with significantly lower rates equal to $a=0.5\;s^{-2}$, $a=1.5\;s^{-2}$, and $a=3.5{\;s}^{-2}$. Typically, the response of the blood in this test combines elastic and thixotropic effects at various levels, primarily depending on the imposed conditions. More specifically, the larger the factor a is, the higher is the applied shear-rate and, consequently, the developed stresses. Initially, in all simulations, blood is found in a stress-free, unperturbed, and fully structured state. In the following figures we show the blood response within a triangular shear step test as function of the transformed time t’. The upward part of the flow curves represents the time from 0 to $t_{max}/2$ while the downward part refers to the time from $t_{max}/2$ to $t_{max}$ with t’ given by:

(24) $$\{\begin{array}{ll} t^{\prime }=t, & t<t_{max}/2\\ t^{\prime }=t_{max}-t, & t\geq t_{max}/2 \end{array}$$

**Figure 18:**
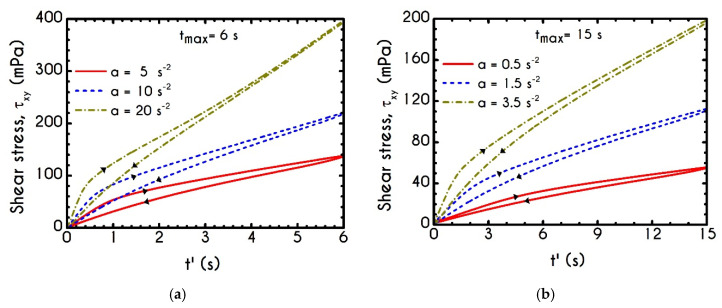
Model predictions for the time evolution of the shear component of the stress tensor during the hysteresis experiment for (**a**) $a=5\;s^{-2}$, $a=10\;s^{-2}$ and $a=20\;s^{-2}$ and maximum duration equal to $t_{max}=\;6\;s$, (**b**) $a=0.5\;s^{-2}$, $a=1.5\;s^{-2}$ and $a=3.5\;s^{-2}$ maximum duration equal to $t_{max}=\;15\;s$.

**Figure 19:**
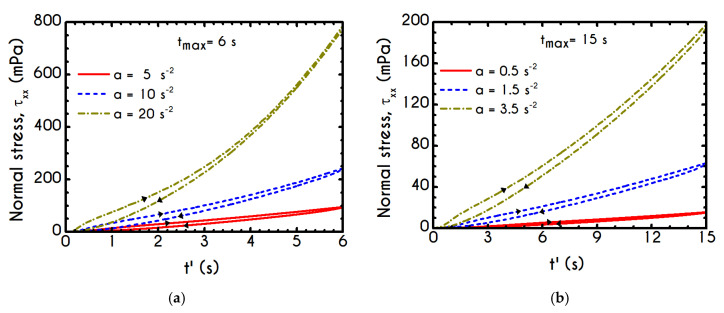
Model predictions for the time evolution of the normal component of the stress tensor during the hysteresis experiment for (**a**) $a=5\;s^{-2}$, $a=10\;s^{-2}$, and $a=20\;s^{-2}$ and maximum duration equal to $t_{max}=\;6\;s$, (**b**) $a=0.5\;s^{-2}$, $a=1.5\;s^{-2},$ and $a=3.5\;s^{-2}$ maximum duration equal to $t_{max}=\;15\;s$.

We choose this visualization to better elucidate the thixotropic effect by forming a close area between the upward and downward parts. Figure 18a presents the shear stress prediction for Case 1, while in Figure 18b, we can see the rheogram of shear stress for Case 2. Obviously, the non-linearity of the flow curves is much more intense than those predicted for normal component of the stress tensor (Figure 19) even for the weaker imposed conditions, namely for the rates equal to $5\;s^{-2}$ with $t_{max}=\;6\;s$, and $0.5\;s^{-2}$ with $t_{max}=\;15\;s$. In comparison with experimental measurements by Bureau et al. [46,89], our TEVP model fitted to the experimental data by McMillan et al. [47], provides qualitatively similar predictions but the hysteresis phenomena are significantly milder. Thus, the enhanced hysteresis of shear-stresses reported in ref. [46,89] could be attributed to pronounced ageing related with those samples, in the case that they had been stored for a long period. 

Rheograms in Figure 19a,b depict the predictions of our model for the evolution of normal component of the stress tensor. As we can see in both those figures, the major contribution in the stress is predominantly elastic. This is more obvious in Case 1 (Figure 19a) than in Case 2 (Figure 19b) as the developed stresses in the system attain higher values, and the initial elastic shock is more intense. Regarding the shear stress, from the Figure 18a we can observe that the maximum predicted values are equal to 136 mPa, 243 mPa, and 788 mPa for rates equal to $a=5{\;s}^{-2}$, $a=10\;s^{-2}$, and $a=20\;s^{-2}$ respectively. Similarly, the corresponding values for the case with $t_{max}=\;15\;s$ are equal *to* 55 mPa, 63 mPa and 197 mPa for rates equal to $a=0.5{\;s}^{-2}$, $a=1.5{\;s}^{-2}$, and $a=3.5\;s^{-2}$ respectively. Although the duration of the latter test is 2.5 times greater than that in Case 1, the predicted stresses within the fluid are much lower than those predicted in Case 1, due to the different selection of a. Obviously, the dominant term in this test is the increase/decrease rate, a. Irrespectively of $t_{max}$, the increase/decrease rate demonstrates a great impact on the thixotropic response of the blood. For both shear and normal components of the stress tensor, an increase in α leads to increase in the thixotropic effect which is obvious by the increase of the enclosed area formed between the flow curves of Figure 18 and Figure 19. Additionally, the higher the increase/decrease rate a is, the more obvious is the non-linearity of the flow curve, revealing the contribution of blood’s thixotropy, as opposed to lower rates for which the rheogram depicts a linear evolution of stress during the experiment. Moreover, the flow curve is distinctly asymmetric, showing a clear dependence on the deformation history. Comparing the effect of thixotropy on the normal stresses to the shear-stresses, we can observe that the hysteresis in normal stresses is significantly weaker. Nevertheless, in both cases, the elastic stress also assumes its maximum at the maximum shear-rate.

Throughout our investigation, our model predicts normal stresses being always lower than the shear counterpart, verifying the majority of the theoretical and limited existing experimental data.

**Figure 20:**
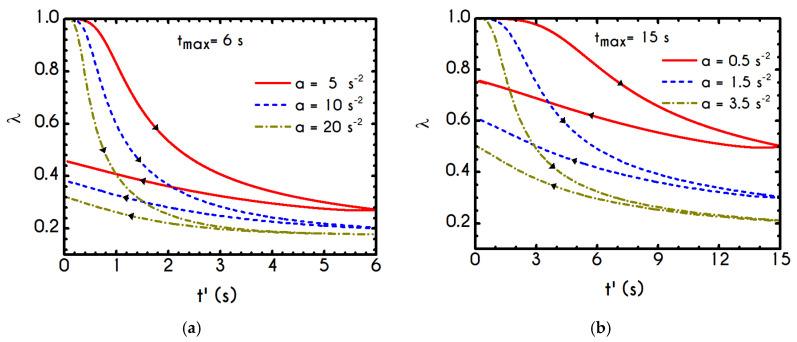
Model predictions for the time evolution of the structure parameter λ during the hysteresis experiment for (**a**) $a=5{\;s}^{-2}$, $a=10\;s^{-2}$ and maximum duration equal to $t_{max}=\;6\;s$, (**b**) $a=0.5\;s^{-2}$, $a=1.5\;s^{-2}$ and $a=3.5\;s^{-2}$ maximum duration equal to $t_{max}=\;15\;s$.

The structure evolution in the fluid in terms of λ and its dependence on the imposed shear-rate and time are illustrated in Figure 20a,b for Case 1 and Case 2, respectively. Beginning with λ(t=0) = 1, the von Mises criterion is not satisfied due to low-stress value, and the structure does not change, because stress is not enough to cause flow. Consequently, there is a short-time area for which blood is maintained at a fully structured state, the size of which dependents apparently on the applied conditions and hence on the combination of a and $t_{max}$. This is less obvious in Case 1, where the applied shear-rate is higher than that predicted for Case 2, and it is gradually faded out as the imposed shear rate is enough to reach the yield strain in a shorter amount of time. This is why the plateau in lambda is wider when a is smaller. Next, the transition to the liquid state begins, and *λ* decreases, as the magnitude of the effective stress changes over time, resulting in the activation of the destruction term in the differential equation for the structure variable. With increasing shear rate, the stresses are gradually increase, while blood is has transitioned to the liquid state. From this point onward, the structure parameter is getting values below unity, indicating a continuous destruction of the rouleaux. Irrespectively of $t_{max}$, for higher rates a, the destruction of the microstructure is quicker and more intense, and the structure parameter decreases earlier and more abruptly, but qualitatively all the curves attain a similar trend. Particularly, blood’s state depicts a reduction form unity to 0.27, 0.21 and 0.18 for rates equal to $a=5\;s^{-2}$, $a=10\;s^{-2}$, and $a=20\;s^{-2}$ respectively. Similarly, the corresponding values for the case with $t_{max}=\;15\;s$ are equal to 0.5, 0.31 and 0.21 for rates equal to $a=0.5\;s^{-2}$, $a=1.5\;s^{-2}$, and $a=3.5\;s^{-2}$ respectively. Meanwhile, the transition to the liquid state signifies the activation of the aging mechanism, which tends to balance destruction mechanism, indicated by the nonlinear reduction of the structure parameter with respect to time. This is a clear manifestation of thixotropy, promoting the reconstruction of the blood and the competitive contribution of the breakdown and build-up terms. Specifically, the final values of blood’s state are equal to 0.45, 0.38 and 0.32 for rates equal to $a=5\;s^{-2}$, $a=10\;s^{-2}$, and $a=20\;s^{-2}$ respectively, while for the Case 2 the corresponding values are equal to 0.76, 0.61 and 0.49 for rates equal to $a=0.5{\;s}^{-2}$, $a=1.5\;s^{-2}$, and $a=3.5\;s^{-2}$ respectively. 

### 4.6. Large Amplitude Oscillatory Shear (LAOS)

To further evaluate the model’s ability to capture the oscillatory shear flow behavior, blood is subjected to the LAOS flow protocol, which has been systematically used in similar studies. Horner and co-workers [92] used a modification of this protocol called UD-LAOS (Unidirectional large amplitude oscillatory shear) as the primary tool to determine the predictive capabilities of their constitutive model, revealing a strong dynamic nature of LAOS especially when transient structural phenomena take place. The flow kinematics may be interpreted as a function of the transient oscillatory strain, which changes continuously with time, and it is described by the expression in Equation (25) where *ω* is the angular frequency of the oscillation and $\gamma_{0}$ is the strain amplitude:

(25) $$\nabla u=\left(\begin{array}{cc} 0 & 0\\ \;\;\;\gamma_{0}\;\omega \;\cos\left(\omega t\right) & 0 \end{array}\right)\;,\;\;t>0\;$$

**Figure 21:**
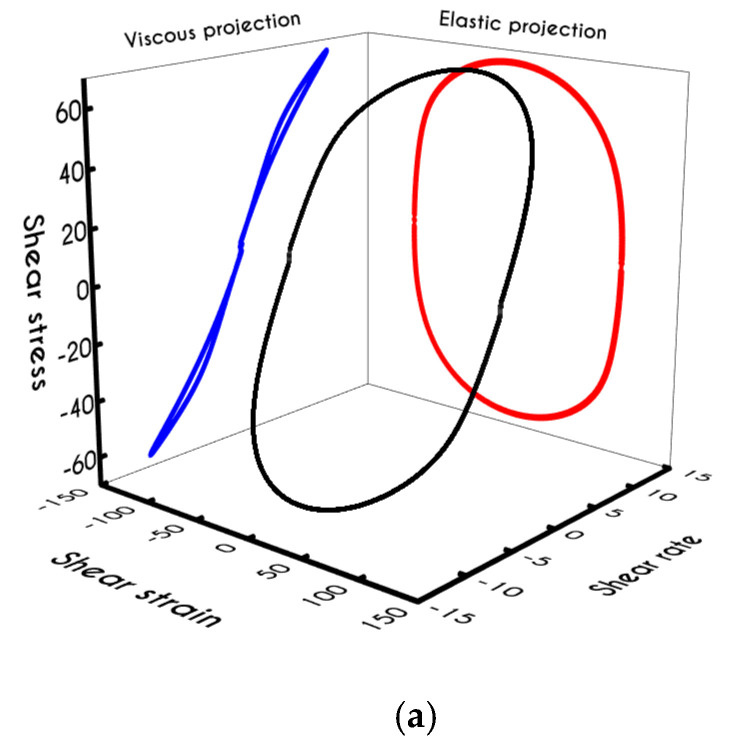

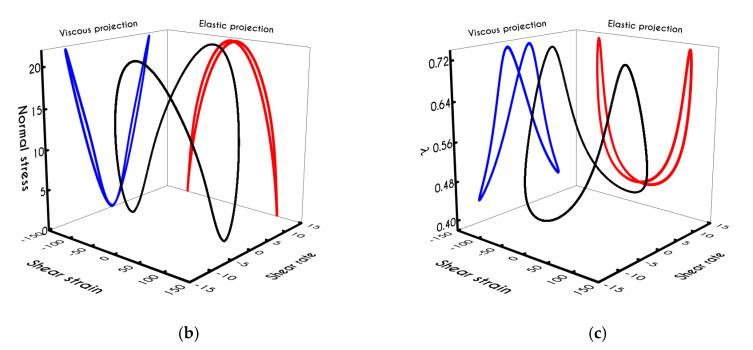
Three dimensional Lissajous–Bowditch (LB) diagram of LAOS test for $\gamma_{0}=100\;$ and ω=0.1 rad/s for the predictions of (**a**) the shear stress $\tau_{xy}$ (in mPa), (**b**) the normal stress $\tau_{xx}$ (in mPa), and (**c**) the structure parameter λ. The shear rate is in $s^{-1}.$

Typically, the response of the fluid in LAOS combines elastic, plastic, and thixotropic effects at various levels, primarily depending on the imposed conditions for $\left(\gamma_{0},\omega \right)$. We perform a parametric analysis for several combinations of $\gamma_{0}$ and ω and assume that blood is initially unperturbed and in a fully structured state. Typically, in the LAOS test, the predictions of stress are represented in three-dimensional space along with the viscous and elastic projections, creating the Lissajous–Bowditch (LB) diagrams, one of which is given in Figure 21. 

The latter corresponds to a strain amplitude and angular frequency equal to $\gamma_{0}=100\;$and ω=0.1 rad/s respectively. The 3D curves and their elastic (stress-strain) and viscous (stress-shear rate) projections demonstrate the typical behavior of blood in that kind of test for the range of the imposed conditions. For the selected frequency and strain amplitude, the material exhibits both elastic and plastic characteristics, which are captured by our model and apart from the major loop that is formed during the oscillatory cycles, we can observe that there are secondary loops in the viscous projection that are related to thixotropic phenomena and stress overshoots, similar to those predicted during the shear startup.

**Figure 22:**
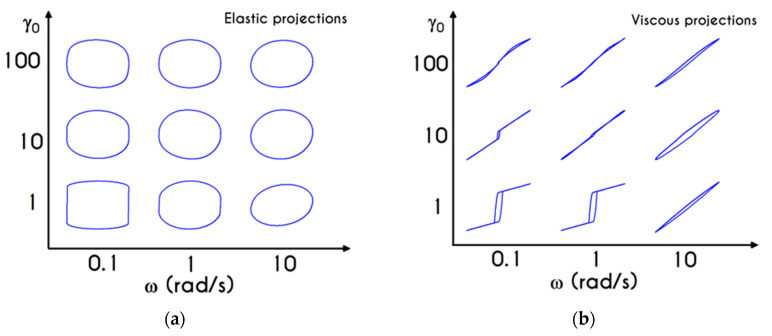
Pipkin diagrams for shear stress $\tau_{xy}$ corresponding to (**a**) Elastic, and (**b**) Viscous projections of LB curves. The results are obtained for $\gamma_{0}$ between 1–100 and ω between 0.1 rad/s–10 rad/s.

**Figure 23:**
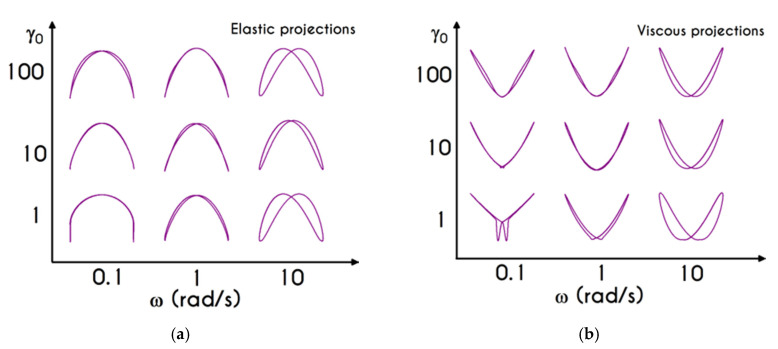
Pipkin diagrams for normal stress $\tau_{xx}$ corresponding to (**a**) Elastic, and (**b**) Viscous projections of LB curves. The results are obtained for $\gamma_{0}$ between 1–100 and ω between 0.1 rad/s–10 rad/.

**Figure 24:**
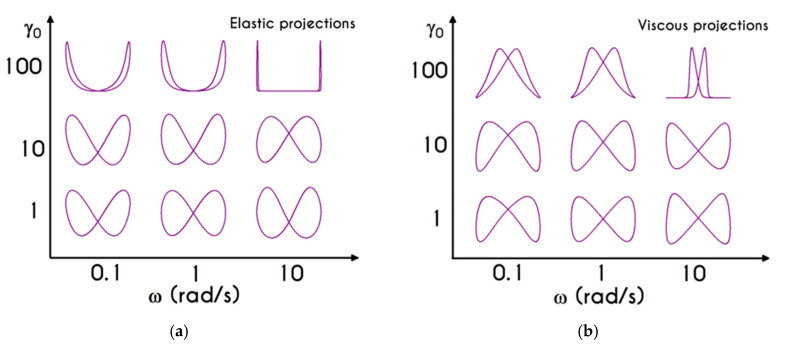
Pipkin diagrams for the structure parameter λ corresponding to (**a**) Elastic, and (**b**) Viscous projections of LB curves. The results are obtained for $\gamma_{0}$ between 1–100 and ω between 0.1 rad/s–10 rad/s.

The stress predictions from the LAOS experiment are represented by the Pipkin diagrams, which involve the elastic and viscous projections, as seen by Figure 22, providing a map for the blood response under widely varying conditions. The elastic projections are in Figure 22a, while the viscous counterpart in Figure 22b. The different rheological behavior of blood provided by these diagrams depends on the amplitude and frequency of the specified case. At high shear strain amplitudes apart from the major loop that is formed during the oscillatory cycles, there is evidence of stress overshoots, similar to those predicted during the startup shear, leading to secondary LAOS loops in the viscous projection that are related to thixotropic effects. The latter phenomena are also observed by Armstrong et al. [93] referring to this as “thixotropic loops,” which indicates that there is structure reforming and breaking down during the oscillation. As the frequency is attaining higher values, this effect becomes less perceived, after which blood demonstrates a weaker structure, and consequently, the rheological response is dominated by viscous forces. A detailed interpretation of these phenomena can be found in the work by de Souza Mendes et al. [94,95]. They investigated the mechanical behavior of materials that possess a microstructure and may exhibit elasto-visco-plastic and thixotropic behavior. In the low strain region, the effects of elasticity are more pronounced as expected. In contrast, for larger strains and frequencies, the response is predominately thixotropic, and the viscous nature of the fluid prevails. For intermediate values ω and $\gamma_{0}$, the microstructural phenomena that take place govern the transitional behavior of the fluid, while the elastic and plastic interplay is still significant. The corresponding Pipkin diagrams for the normal stress $\tau_{xx}$ and the structure parameter *λ* are shown in Figure 23 and Figure 24, respectively.

### 4.7. Uniaxial Elongation

The current test refers to a protocol where the blood sample is subjected to an extensional flow, but it is a topic that has not received appropriate attention compared to shear flows or the aforementioned transient tests. Experimental data or theoretical predictions of blood rheology for this test are quite limited, and this test needs further examination to become useful for the evaluation of the model’s rheological parameters and hence the predictive capability of our model. Indicatively, Kolbasov et al. [96] prepared uniaxial elongation experiments and clearly distinguished the viscoelastic behavior of blood characterizing, besides the relaxation time scale and the magnitude of the elongational viscosity of blood. A recent study was also published by Pihno et al. [97]. However, the available investigations are extremely limited, and hence we cannot make a comparison. The flow kinematics of uniaxial elongation is given by the following expression where ${\dot{\varepsilon }}_{0}$ is the elongation rate:

(26) $$\nabla u=\left(\begin{array}{ccc} {\dot{\varepsilon }}_{0} & 0 & 0\\ 0 & -{\dot{\varepsilon }}_{0}/2 & 0\\ 0 & 0 & -{\dot{\varepsilon }}_{0}/2 \end{array}\right)\;,\;\;t>0$$

In the absence of experimental data, we present our model predictions for the uniaxial elongation flow for blood. The implementation is separated into a steady-state analysis and a transient one regarding the imposed elongation rate. For all simulations, we adopt the assumption that initially, the blood is unperturbed and in fully structured state yielding that stresses are zero and λ is equal to unity at t=0. 

**Figure 25:**
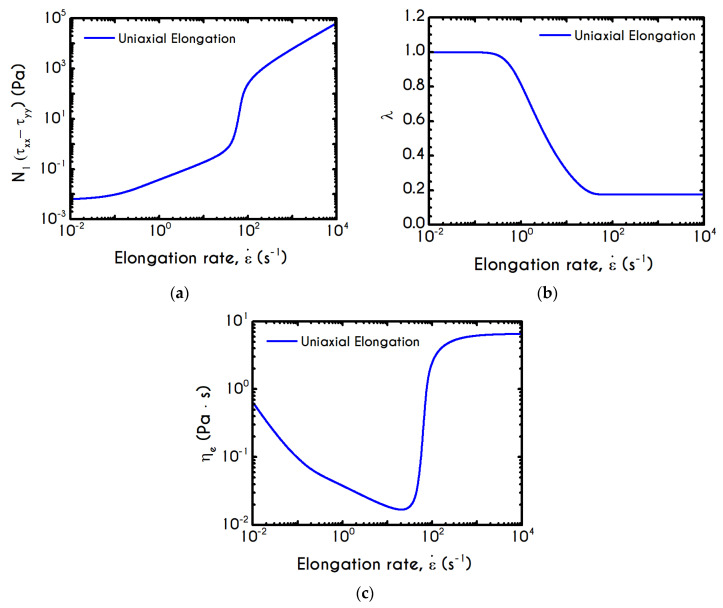
(**a**) Steady normal stress difference $N_{1}\left(\tau_{xx}-\;\tau_{yy}\right)$ with respect to elongation rate, (**b**) structure parameter λ and (**c**) extensional viscosity $\eta_{e}\;\left(=N_{1}/\dot{\varepsilon }\right)$ as a function of the imposed elongation rate in uniaxial extension flow.

Figure 25a demonstrates the predictions of the TEVP model for the steady normal stress difference $N_{1}\left(\tau_{xx}-\;\tau_{yy}\right)$ as a function of the elongation rate ${\dot{\varepsilon }}_{0}$. Our model predicts that the normal stress difference increases non-linearly and monotonically with elongation rate. From ${\dot{\varepsilon }}_{0}=0.01\;s^{-1}\;$to ${\dot{\varepsilon }}_{0}=30\;s^{-1}$ we observe a gradual increase in normal stress difference while for elongation rates higher than 30$\;s^{-1}$, the prediction of $N_{1}$ follows a steep increment. Additionally, the extensional yield stress for low elongation rate is equal to 3.8 mPa, while in steady shear flow, our model predicts a yield stress equal to 3.51 mPa. Since no extensional experiments exist for blood, we cannot compare directly our predictions and thus assess the validity of our predictions under uniaxial elongation. Nevertheless, since blood is a suspension of elastic particles (RBCs) in a viscoelastic solvent (plasma), we can make some theoretical considerations regarding the quantitative predictions of our model in uniaxial elongation. The small plateau that arises in the plastic regime is in accordance with recent experimental measurements of “the normal yield stress in elongation” in various jammed systems [98] and is expected to be present in extensional experiments with blood. The subsequent regime, where $N_{1}$ rises smoothly, corresponds to the onset of viscous fw and thixotropic phenomena related to the destruction of the rouleaux. Finally, at extreme elongation rates we can observe that $N_{1}$ rises sharply before attaining a constant slope. Although in such high extension rates the blood is found in a fully destructed state, it is obvious that this strain-hardening behavior is directly related to the viscoelastic dilute, viz. blood plasma. The extensional flow field in this regime is so intense (*ε* > 50 s^−1^) that is capable of orienting the deformable cellular components of blood and unfolding the high molecular weight proteins (fibrinogen, G-immunoglobulin, and albumin) that are diluted inside plasma, leading to this extra viscoelastic resistance in elongation. For the sake of convenience, we provide a function given by Equation (27) which reproduces the predictions of the first normal difference $N_{1}$ with respect to elongation rate $\dot{\varepsilon }$ of the Figure 25a as:

(27) $$N_{1}\left(\dot{\varepsilon }\right)\;=\;\frac{A_{1}+A_{2}\dot{\varepsilon }}{1+A_{3}\dot{\varepsilon }+A_{4}{\dot{\varepsilon }}^{2}}$$

We also provide an analytical function for the variation of extensional viscosity $\eta_{e}$ with $\dot{\varepsilon }$ as:

(28) $$\eta_{e}\left(\dot{\varepsilon }\right)=\;\frac{N_{1}\left(\dot{\varepsilon }\right)\;}{\dot{\varepsilon }}=\frac{1}{\dot{\varepsilon }}\frac{A_{1}+A_{2}\dot{\varepsilon }}{1+A_{3}\dot{\varepsilon }+A_{4}{\dot{\varepsilon }}^{2}}$$

The corresponding parameters are illustrated in Table 3. 

Figure 25b demonstrates the steady state values of the microstructural parameter λ as a function of the imposed elongation rate ${\dot{\varepsilon }}_{0}$. For elongation rates up to ${\dot{\varepsilon }}_{0}=0.02\;s^{-1}$ the blood state remains in a fully structured condition, i.e., λ = 1, and then it experiences an abrupt reduction to λ = 0.01 at an elongation rate equal to ${\dot{\varepsilon }}_{0}\;=\;22\;s^{-1}$. After this point, the state of the blood cannot present further deconstruction, and thus we do not observe any changes in *λ*. From Figure 25c we can observe the extensional viscosity $\eta_{e}\;\left(N_{1}/\dot{\varepsilon }\right)$ as a function of the imposed elongation rate. As the elongation rates increases, the structural clustering is disintegrated and consequently we observe a thinning behavior. For elongation rates ${\dot{\varepsilon }}_{0}>50\;s^{-1}$, the deconstruction of the blood has reached an asymptotic behavior (as we can see from Figure 25b) and we observe a thickening behavior of the extensional viscosity. This is originated from the fact that we have fitted the high-shear rate plateau of shear viscosity and hence we include the contribution of the plasma viscoelasticity. Consequently, we can assert that blood has an extensional thickening behavior. 

**Figure 26:**
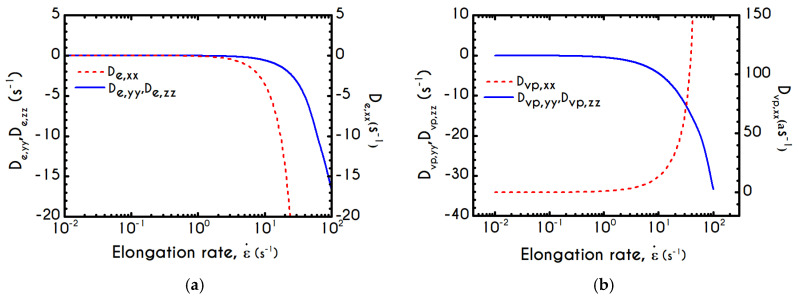
(**a**) Elastic $D_{e,xx}$, $D_{e,yy}$ and $D_{e,zz}$ and (**b**) viscoplastic $D_{e,xx}$, $D_{e,yy}$ and $D_{e,zz}$ projections of the deformation rate tensor as a function of uniaxial elongation $\dot{\varepsilon }$.

At this point, it is very interesting to investigate the role of elastic and viscous contribution to the total deformation rate as a function of the elongation rate in steady-state uniaxial extension flows. Particularly, Figure 26 illustrates the xx−, yy− and zz− (yy− and zz− are equal) contributions of elastic (Figure 26a) and viscous (Figure 26b) components as a function of the imposed elongation rate ${\dot{\varepsilon }}_{0}$ using the parametric set extracted from the fitting process on blood experimental date reported in [47]. The elastic response of blood in this test is evaluated through a double-y-axis graph due to different scaling, as depicted by Figure 26a. Although qualitatively, xx− and yy− contributions are similar, they differ significantly in magnitude. At low elongation rates, the developed shear rates present in the system are quite insignificant, but for higher rates, our model predicts an abrupt increase in its absolute value.

**Figure 27:**
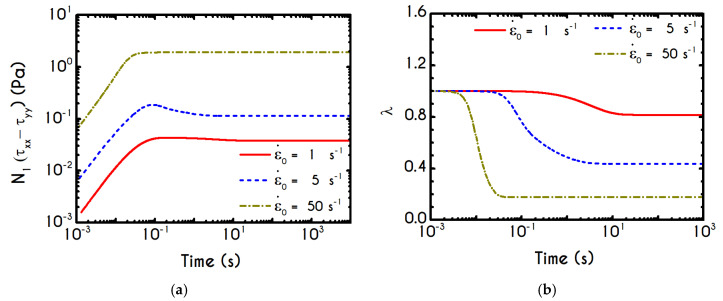
Transient predictions of uniaxial elongation experiment for different elongation rates ${\dot{\varepsilon }}_{0}$ equal to $1{\;s}^{-1}$, $5{\;s}^{-1}$ and $50{\;s}^{-1}$ for (**a**) the steady normal stress difference $N_{1}\left(\tau_{xx}-\;\tau_{yy}\right)$, and (**b**) structure parameter λ.

Next, in Figure 27a, we examine the evolution of the normal stress differences for three different elongation rates ${\dot{\varepsilon }}_{0}\;$equal to $1{\;s}^{-1}$, $5{\;s}^{-1}$, and $50{\;s}^{-1}$. The initial startup is governed by an elastic response where the material behaves as a Neo-Hookean solid during stretching. Particularly, for the highest shear rate imposed, a high overshoot in the stress curve is observed at t=0.0442 s which is equal to $N_{1}=\;1.91\;\mathrm{Pa}$ and tends to be balanced before the steady-state is achieved. In lower elongation rates, this overshoot is significantly weaker, and in the case of the lowest value of elongation rate, it is almost absent. 

In Figure 27b we can observe the evolution of the instantaneous state of blood during the uniaxial elongation test for the aforementioned elongation rates. As expected, the microstructural parameter illustrates a strong dependence on the imposed elongation rate ${\dot{\varepsilon }}_{0}\;$and the steady state for each case is achieved through different dynamics. In all cases, for early times of the experiment the blood remains at a fully structured state, then it experiences an abrupt disintegration within a specific time interval and eventually the microstructural parameter achieves a steady state. Indicatively, the time after which blood’s state starts to breakdown is 0.22 s, 0.03 s and 0.004 s for $1{\;s}^{-1}$, $5{\;s}^{-1}$ and $50{\;s}^{-1}$ respectively. As the elongation rate increases, the state of blood depicts an earlier deconstruction, the reduction is steeper, and the final aggregation level is lower. 

**Figure 28:**
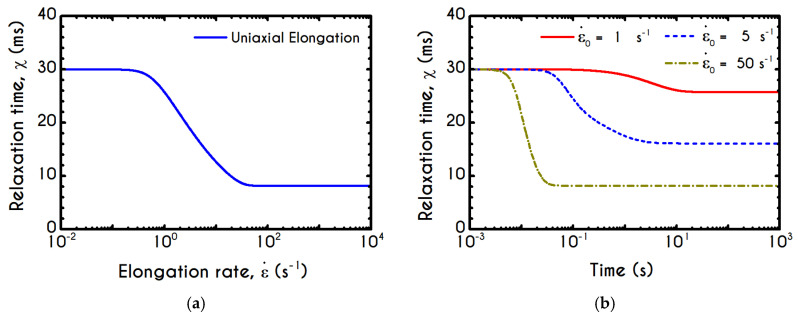
Steady (**a**) and Transient predictions (**b**) of the uniaxial elongation experiment for different elongation rates ${\dot{\varepsilon }}_{0}$ equal to $1\;s^{-1}$, $5{\;s}^{-1}$, and $50{\;s}^{-1}$.

From Figure 28a,b we can observe the prediction of the blood relaxation time χ in steady and transient uniaxial elongation test, respectively. Regarding the steady state results (Figure 28a), we can distinguish three responses of the blood as a function of the imposed elongation rate ${\dot{\varepsilon }}_{0}$. From ${\dot{\varepsilon }}_{0}=0.24{\;s}^{-1}$ to ${\dot{\varepsilon }}_{0}=440{\;s}^{-1}$ the relaxation time experiences a significant reduction from χ=3.14 ms to χ=0.97 ms respectively. Outside from the latter range of ${\dot{\varepsilon }}_{0}$ an upper and a lower plateau indicating that the state of blood depicts no changes with elongation rates and thus the plastic viscosity $\eta_{t}$ is constant, corresponding to that of plasma [39]. The evolution of the relaxation time is illustrated in Figure 28b for three elongation rates ${\dot{\varepsilon }}_{0}\;$equal to $1\;s^{-1}$, $5{\;s}^{-1}$, and $50{\;s}^{-1}$. The transient results of the relaxation time χ follow similar dynamics with those predicted for the microstructural parameter λ of the Figure 27b. Essentially, the plastic viscosity is directly associated with the instantaneous state of blood through Equation (13) and thus it is a thixotropic property. Similar with the λ evolution, the higher the imposed elongation rate, the steeper the reduction of the relaxation time as well as the lower the final value of χ.

**Figure 29:**
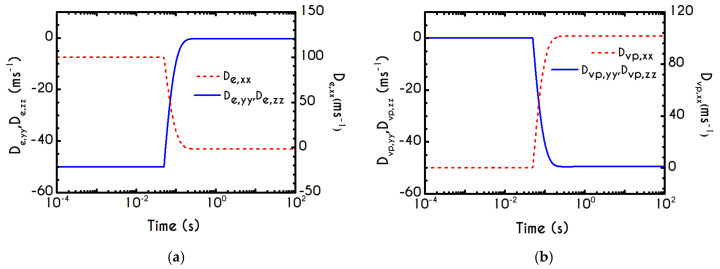
(**a**) Elastic, $D_{e,yy}$ and $D_{e,zz}$ and (**b**) viscoplastic $D_{vp,xx}$, $D_{vp,yy}$ and $D_{vp,zz}$ projections of the deformation rate tensor as a function of time for the uniaxial elongation experiment for ${\dot{\varepsilon }}_{0}=0.1\;s^{-1}$.

**Figure 30:**
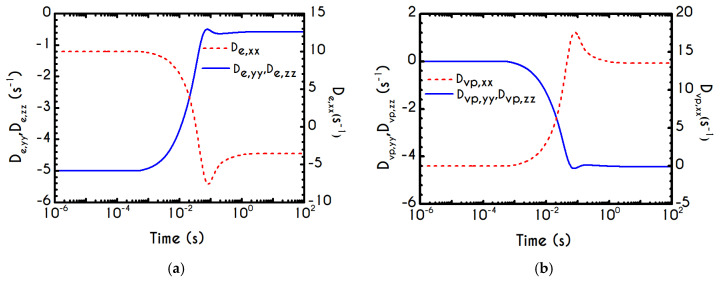
(**a**) Elastic $D_{e,xx}$, $D_{e,yy}$ and $D_{e,zz}$ and (**b**) vsiscoplastic $D_{vp,xx}$, $D_{vp,yy}$ and $D_{vp,zz}$ projections of the deformation rate tensor as a function of time for the uniaxial elongation experiment for ${\dot{\varepsilon }}_{0}=10\;s^{-1}$.

To further study the rheological behavior of blood and consequently to highlight the internal mechanisms, we illustrate the transient predictions of our model for the elastic and viscous contributions of the deformation rate tensor as functions of time for two distinct elongation rates ${\dot{\varepsilon }}_{0}$. For the sake of convenience, the obtained results are plotted in double-*y*-axis diagrams due to different scaling, as depicted by Figure 29 and Figure 30 for elongation rates equal to ${\dot{\varepsilon }}_{0}=0.1\;s^{-1}$ and $10\;s^{-1}$ respectively. As it is expected, the effect of the imposed elongation rate ${\dot{\varepsilon }}_{0}$ on the blood response is reasonable. In the case with ${\dot{\varepsilon }}_{0}=0.1\;s^{-1}$ viscous contribution is initially zero Figure 29b due to fact the blood response is predominantly elastic. This can be proven by Figure 29a where elastic contribution is smoothly increased until a steady state is achieved. On the contrary, for a 100 times greater elongation rate, the blood response is smoother than the previous one. We can observe from Figure 30a the initial overshoot of elastic contribution until the steady state is achieved at time equal to t = 0.1 s for both xx− and yy− (or zz−) components. Moreover, the imposition of a high elongation rate will result to overcome the yield stress in a shorter amount of time, and this is why we do not observe an initial zero-plateau at the viscous contribution in Figure 30b as it was depicted by Figure 29b. For the viscous contribution we can observe a more intense overshoot than the elastic one while the steady state is achieved at the same time.

## 5. Conclusions

In this work, we modified the constitutive law for blood flow modeling, introducing a thixotropic elasto-viscoplastic formulation. The proposed model was based on the formulation of Varchanis et al. [62], which incorporated thixotropy, elasticity and viscoplasticity following an additive elastoplastic decomposition. It was able to capture all the essential steady and transient experimental data. It encompassed all the crucial aspects of blood rheology, including yield stress, potential thixotropic effects, and elasticity, which are associated with the aggregation/disaggregation of the erythrocytes, as well as the viscoelastic contribution to whole blood that is induced by the interaction of the macroscopic flow with the diluted proteins in blood plasma. The latter features are accompanied by the introduction of a microstructural state indicator that dynamically responded depending on the current rate of shear and stress present in the system.

We mainly presented the predictive capability of the proposed model in various steady and transient rheometric flows, including startup tests, intermittent step tests, startup cessation, and triangular tests. The model predictions of startup flows underlined the importance of both elasticity and thixotropy. The linear dependence of stress on the imposed strain observed at the beginning of the test suggested that elastic forces are developed in the material. In contrast, the formation of intense overshoots and the subsequent stress relaxation elucidated the thixotropic response. This phenomenon was quantified by the thixotropic index, which indicated a higher value for a more intense thixotropic response. Next, the input of startup followed by a cessation protocol was examined, which provided a more complex response of the material in terms of the structure parameter, depending on the imposed flow conditions. The higher the imposed shear rate was, the shorter was the period in which the material’s state remained in its solid state. After this point, the stresses developed in the blood satisfied the von Mises criterion, and the microstructural clustering began to collapse until the cessation of the flow, which caused an abrupt increase on λ, and hence the aggregation of RBCs led to a fully structured state. The intermittent test revealed that the thixotropic time scale governs the elasto-thixotropic response of blood under flow rearrangements. The size of the observed overshoots was affected by the duration of cessation between two shear impositions, where a small Δt indicated a weaker elastic response of the material for the sequential process. The predictive capability of the model was also evaluated in triangular step experiments for different increase shear rates. In accordance with respective experiments with blood, our model predicted hysteretic loops associated with the thixotropic nature of blood. Apart from the integrated modeling of blood’s rheological complexity, our model is capable of providing the relation between the stress and the deformation rate tensors in realistic flow simulations of blood, where the momentum and mass conservation laws are solved coupled with the constitutive equation; this is very important as many models that describe the rheology of blood are presented in a simple scalar, shear stress formalism [99,100,101]. Finally, the nonlinear dependence of the material parameters of the model on the structure parameter and the first invariant of the stress tensor allowed for a single mode approach, which can at the same time capture elastic, plastic, viscous, thixotropic and viscoelastic effects in a wide range of deformation rates and provide a low computational cost compared to another TEVP models. 

## Figures and Tables

**Table 1:** Fitted rheological parameters of TEVP model on steady and transient experimental data reported in [47,48].

Parameters	Units	Subject of [47]	Donor 1 of [48]
*G*	Pa	0.382	0.15
$\eta_{0}$	Pa·s	0.012	0.035
$\tau_{y}$	Pa	0.0035	0.006
$\varepsilon_{PTT}$	−	0.001	0.001
$k_{1}$	$s^{-1}$	0.0918	0.092
$k_{2}$	$s^{n_{1}-1}$	7.249	0.45
$k_{3}$	$s^{n_{2}-1}$	6974.9	1720
$n_{1}$	−	3.03	1.67
$n_{2}$	−	4.068	3.41
$n_{3}$	−	3.03	1.67
$m_{1}$	−	0.701	0.91

**Table 2:** Fitted parameters of Equation (15) for the description of *λ* as a function of shear rate.

Parameters	Units	Values
A	−	1.0
B	−	0.1763
k	$s^{-1}$	4.795
n	−	1.262

**Table 3:** Fitted parameters of Equation (27) for the description of $N_{1}$ and $\eta_{e}$ as functions of elongation rate $\dot{\varepsilon }$ for subject in [47].

Parameters	Units	Values
$A_{1}$	Pa	−1.69
$A_{2}$	Pa·s	1.30
$A_{3}$	s	−0.427
$A_{4}$	$s^{2}$	0.0931

## Appendix A. Inelastic Models

Here we determine the parameters for the other generalized Newtonian models from the literature data. The corresponding flow curves are given in Figure 6a. The majority of the blood rheology investigations are focused on steady-state experiments, and their models are valid only for the description of shear-thinning or yield stress. Among the most known to adequately describe the apparent viscosity as a function of shear rate are those of Cross [100] and Carreau-Yasuda et al. [102]. The latter model is broadly used to describe the shear-thinning behavior of blood [103,104], and the apparent viscosity as a function of shear rate is given by:

(A1) $$\eta \left(\dot{\gamma }\right)=\eta_{\infty ,cy}+\left(\eta_{0,cy}-\eta_{\infty ,cy}\right){\left[1+{\left(\lambda_{cy}\dot{\gamma }\right)}^{\alpha_{cy}}\right]}^{\frac{n_{cy}-1}{2}}\;$$

where $\eta_{0,cy}$ and $\eta_{\infty ,cy}$ are the zero-shear rate viscosity and the viscosity at high shear rates respectively, while $\lambda_{cy},\alpha_{cy}$ and $n_{cy}$ are adjustable parameters of the model. Table A1 contains the aforementioned rheological parameters, which are evaluated by a non-linear fitting on apparent viscosity experimental data reported in [47]. Another generalized Newtonian model is that of Cross which has also gained considerable popularity, and the apparent viscosity is given by:

(A2) $$\eta \left(\dot{\gamma }\right)=\eta_{\infty ,c}+\left(\eta_{0,c}-\eta_{\infty ,c}\right)\left[\frac{1}{1+\;{\left(\lambda_{c}\dot{\gamma }\right)}^{m_{c}}}\right]$$

where $\eta_{0,c}$ and $\eta_{\infty ,c}$ are the zero-shear rate viscosity and the viscosity at high shear rates respectively, while $\lambda_{c}$ and $m_{c}$ are adjustable parameters of the model. For this model, we prepared two fittings of distinct experimental data for the apparent viscosity. Except for McMillan’s experimental data, we also make use of the experimental observations of Brooks et al. [79] for the same hematocrit. The adjustable rheological parameters referring to the experimental data of McMillan and Brooks are given in Table A2 and Table A3, respectively. Another widely used model is that of Casson [101] which encompasses the feature of yield stress along with the shear thinning description and the expression for the developed stress is given by:

(A3) $$\sqrt{\tau_{xy}\left(\dot{\gamma }\right)}=\sqrt{\tau_{y}}+\;\sqrt{\mu }\sqrt{\dot{\gamma }}$$

where $\tau_{y}$ and μ are the yield stress and the viscosity, respectively, while they both are fitted to experimental data of McMillan et al. [47] and tabulated in Table A4.

**Table A1:** Fitted rheological parameters of the Carreau-Yasuda model on steady experimental data reported in [47].

Parameters	Units	Values
$\eta_{0,cy}$	Pa·s	0.10773
$\eta_{\infty ,cy}$	Pa·s	0.0040
$\lambda_{cy}$	s	20.112
$\alpha_{cy}$	−	2.2169
$n_{cy}$	−	0.36236

**Table A2:** Fitted rheological parameters of the Cross model on steady experimental data reported in [47].

Parameters	Units	Values
$\eta_{0,c}$	Pa·s	0.2504
$\eta_{\infty ,c}$	Pa·s	0.0034
$\lambda_{c}$	s	51.91
$m_{c}$	−	0.729

**Table A3:** Fitted rheological parameters of the Cross model on steady experimental data reported in [79].

Parameters	Units	Values
$\eta_{0,c}$	Pa·s	0.3079
$\eta_{\infty ,c}$	Pa·s	0.0034
$\lambda_{c}$	s	15
$m_{c}$	−	0.539

**Table A4:** Fitted rheological parameters of the Casson model on steady experimental data reported in [47].

Parameters	Units	Values
$\tau_{y}$	Pa	0.0033
μ	Pa·s	0.00389

It is worth mentioning the capability of our model against other generalized Newtonian or non-Newtonian models regarding their characteristics. There are many investigations that used generalized Newtonian models [7,101,105], mostly accounting for the shear-thinning response, but only a few have used the TEVP model [49,59]. For the first time, we present a tensorial constitutive model that accounts for blood viscoplasticity at stasis, thixotropy, shear thinning for the whole range of shear rates, extensional and shear viscoelasticity, viscous dissipation and viscoelastic matrix contribution in a fully coupled manner. Table A5 provides a comparison of the rheological characteristics of our model, the modified Casson model of [101], the model of Owens [54] the model of Cross [100] and that of Apostolidis et al. [57].

**Table A5:** Basic rheological features of the modified Casson model of [101], the model of Owens [54], and the Cross model [100], the model of Apostolidis et al. [57] and the TEVP model.

Characteristics	Modified Casson [101]	Owens [54]	Cross [100]	Apostolidis [57]	TEVP
tensorial	–	✓	–	–	✓
shear-thinning	✓	✓	✓	✓	✓
plasticity	✓	–	–	✓	✓
viscoelasticity	–	✓	–	✓	✓
thixotropy	–	✓	–	✓	✓

## Appendix B. Reduced Form of the Equations

In the “Constitutive Modelling” section we presented the tensorial form of the model. Here we provide the set of the reduced form of the Equations (3)–(6) for each flow kinematics, which forms a set of ODEs solved by the Runge-Kutta method. The equations for the startup, shear cessation and rectangular shear-step tests are identical, and they only differ in the time/duration of the shear imposition. Using Equation (16) we obtain the following system of equations:

(A4) $$\;\;f\left[tr\left(\tau_{eff}\right)\right]\max\left(0,\frac{\sigma_{eff}-\;\tau_{y}}{2\;\eta_{t}\;\sigma_{eff}}\right)\tau_{xx}+\frac{1}{2\;G}\;\left[\frac{d\tau_{xx}}{dt}-2\tau_{xy}\dot{\gamma }\right]=0$$

(A5) $$\;\;f\left[tr\left(\tau_{eff}\right)\right]\max\left(0,\frac{\sigma_{eff}-\;\tau_{y}}{2\;\eta_{t}\;\sigma_{eff}}\right)\tau_{xy}+\frac{1}{2\;G}\;\frac{d\tau_{xy}}{dt}-\frac{1}{2}\dot{\gamma }=0$$

(A6) $$\;\;\frac{d\lambda }{dt}=\left(k_{1}+k_{2}\max{\left(0,\sigma_{eff}-\;\tau_{y}\right)}^{n_{1}}\right)\left(1-\lambda \right)-k_{3}\max{\left(0,\sigma_{eff}-\;\tau_{y}\right)}^{n_{2}}\lambda^{n_{3}}$$

where Equations (A4) and (A5) refer to the unknowns $\tau_{xx}$ and $\tau_{xy}$ respectively. For the startup experiment the above system is solved for an imposed shear rate equal to $\dot{\gamma }={\dot{\gamma }}_{0}$ for t≥0 until a steady state is achieved. For the shear cessation experiment we solve the above system according to:

(A7) {γ˙=γ˙0  ,  t<tces  γ˙=0    ,  t≥tces 

while for the rectangular shear-step test we solve Equations (A4) and (A5) for:

(A8) {γ˙=0     ,                  t1≤t≤t2∪  t≥t3  γ˙=γ˙0     ,           0<t<t1∪  t2<t<t3 

In the triangular shear-step test, $\dot{\gamma }$ in Equations (A4) and (A5) is replaced by the product at as:

(A9) $$\;\;f\left[tr\left(\tau_{eff}\right)\right]\max\left(0,\frac{\sigma_{eff}-\;\tau_{y}}{2\;\eta_{t}\;\sigma_{eff}}\right)\tau_{xx}+\frac{1}{2\;G}\;\left[\frac{d\tau_{xx}}{dt}-2\tau_{xy}at\right]=0$$

(A10) $$\;\;f\left[tr\left(\tau_{eff}\right)\right]\max\left(0,\frac{\sigma_{eff}-\;\tau_{y}}{2\;\eta_{t}\;\sigma_{eff}}\right)\tau_{xy}+\frac{1}{2\;G}\;\frac{d\tau_{xy}}{dt}-\frac{1}{2}at=0$$

(A11) $$\;\;\frac{d\lambda }{dt}=\left(k_{1}+k_{2}\max{\left(0,\sigma_{eff}-\;\tau_{y}\right)}^{n_{1}}\right)\left(1-\lambda \right)-k_{3}\max{\left(0,\sigma_{eff}-\;\tau_{y}\right)}^{n_{2}}\lambda^{n_{3}}$$

where a is a negative/positive rate and t is the time. We then solve the corresponding system of Equations (A9) and (A10) according to:

(A12) {α=a0  ,  t<tmax/2  α=−a0  ,  t≥tmax/2 

where $a_{0}$ is the magnitude of the rate of increase/decrease in shear rate measured in $s^{-2}$. In LAOS we solve the following system:

(A13) $$\;\;f\left[tr\left(\tau_{eff}\right)\right]\max\left(0,\frac{\sigma_{eff}-\;\tau_{y}}{2\;\eta_{t}\;\sigma_{eff}}\right)\tau_{xx}+\frac{1}{2\;G}\;\left[\frac{d\tau_{xx}}{dt}-2\tau_{xy}\gamma \;\omega \;\cos\left(\omega t\right)\right]=0$$

(A14) $$\;\;f\left[tr\left(\tau_{eff}\right)\right]\max\left(0,\frac{\sigma_{eff}-\;\tau_{y}}{2\;\eta_{t}\;\sigma_{eff}}\right)\tau_{xy}+\frac{1}{2\;G}\;\frac{d\tau_{xy}}{dt}-\frac{1}{2}\gamma \;\omega \;\cos\left(\omega t\right)=0$$

(A15) $$\;\;\frac{d\lambda }{dt}=\left(k_{1}+k_{2}\max{\left(0,\sigma_{eff}-\;\tau_{y}\right)}^{n_{1}}\right)\left(1-\lambda \right)-k_{3}\max{\left(0,\sigma_{eff}-\;\tau_{y}\right)}^{n_{2}}\lambda^{n_{3}}$$

with $\omega =\omega_{0}$ and $\gamma =\gamma_{0}$ for t≥0. Finally, in uniaxial extension test we solve the following system:

(A16) $$\;\;f\left[tr\left(\tau_{eff}\right)\right]\max\left(0,\frac{\sigma_{eff}-\;\tau_{y}}{2\;\eta_{t}\;\sigma_{eff}}\right)\tau_{xx}+\frac{1}{2\;G}\;\left[\frac{d\tau_{xx}}{dt}-2\tau_{xx}\dot{\varepsilon }\;\right]-\dot{\varepsilon }=0$$

(A17) $$\;\;f\left[tr\left(\tau_{eff}\right)\right]\max\left(0,\frac{\sigma_{eff}-\;\tau_{y}}{2\;\eta_{t}\;\sigma_{eff}}\right)\tau_{yy}+\frac{1}{2\;G}\;\left[\frac{d\tau_{yy}}{dt}+\tau_{yy}\dot{\varepsilon }\;\right]+\frac{\dot{\varepsilon }}{2}=0$$

(A18) $$\;\;f\left[tr\left(\tau_{eff}\right)\right]\max\left(0,\frac{\sigma_{eff}-\;\tau_{y}}{2\;\eta_{t}\;\sigma_{eff}}\right)\tau_{zz}+\frac{1}{2\;G}\;\left[\frac{d\tau_{zz}}{dt}+\tau_{zz}\dot{\varepsilon }\;\right]+\frac{\dot{\varepsilon }}{2}=0$$

(A19) $$\;\;\frac{d\lambda }{dt}=\left(k_{1}+k_{2}\max{\left(0,\sigma_{eff}-\;\tau_{y}\right)}^{n_{1}}\right)\left(1-\lambda \right)-k_{3}\max{\left(0,\sigma_{eff}-\;\tau_{y}\right)}^{n_{2}}\lambda^{n_{3}}$$

with $\dot{\varepsilon }={\dot{\varepsilon }}_{0}$ for t≥0.
